# Complete genome characterization of foot-and-mouth disease virus My-466 belonging to the novel lineage O/ME-SA/SA-2018

**DOI:** 10.1016/j.heliyon.2024.e26716

**Published:** 2024-02-20

**Authors:** Humaira Anjume, Kazi Alamgir Hossain, Anamica Hossain, M. Anwar Hossain, Munawar Sultana

**Affiliations:** Department of Microbiology, University of Dhaka, Dhaka, 1000, Bangladesh

**Keywords:** Foot-and-mouth disease (FMD), Foot-and-mouth disease virus (FMDV), O/ME-SA/SA-2018, Novel sublineage, First complete genome

## Abstract

Foot-and-mouth disease virus (FMDV), the causative agent of the foot-and-mouth disease of cattle population possesses a rapid evolutionary rate. In Bangladesh, the first circulation of the O/ME-SA/SA-2018 lineage as a novel sublineage, MYMBD21 was reported from our laboratory. The first whole genome sequence of an isolate, BAN/MY/My-466/2021 (shortly named My-466) of the SA-2018 lineage is characterized and represented in this study. The genome is 8216 nucleotides long with 6996 nucleotides open reading frame flanked by 5ꞌ UTR (1–1100) and 3ꞌ UTR (8097–8216). VP1 was found to be highly variable among the structural proteins with crucial mutations in the major antigenic region, G-H loop. Structural variations of the VP1 against both field and proposed local vaccine strains were evidenced by the G-H loop displacement in a superimposed 3D model. The complete genome information of the isolate would be valuable for undertaking proper control measures needed to limit the spread of the newly emerged FMDV strain.

## Highlights

• We provided the first complete genome sequence analysis of the novel foot-and-mouth disease virus lineage, O/ME-SA/SA-2018.
• The Lpro region demonstrated the highest nucleotide and protein variability among the coding sequences tested.
• A comparative sequence analysis against vaccine strains detected VP1 as the most variable capsid protein.

## 1 Introduction

The disease threatening the health and lives of more than 70 cloven-hoofed animals, including pigs, cattle, sheep, and goats all across the world is none other than the foot-and-mouth disease (FMD) [1]. The causative viral agent belongs to the genus *Aphthovirus* within the family *Picornaviridae* [[1], [2], [3], [4]]. It not only hampers animal welfare and production but also has devastating socio-economic effects in endemic countries, particularly in Asia and Africa and parts of South America where a huge portion of the national economy depends on the cattle industry. Foot-and-mouth disease virus (FMDV) being an RNA virus lacks the proofreading activity of the polymerase resulting in higher mutational frequency that generates a wide variety of strains within the FMDV population [5,6].

The genome of FMDV is a positive-sense single-stranded RNA (>8 kb in length) with a single large open reading frame (ORF) that is flanked by highly structured 5′ and 3′ untranslated regions (5′ UTR and 3′UTR). The genome is enclosed within an icosahedral capsid constituting 60 identical protomers of one copy of each of the four structural proteins, VP1, VP2, VP3, and VP4 [1,5]. The 5ꞌ UTR includes several distinct structural elements including S-fragment, a poly(C) tract (Cn), 3 or 4 pseudoknots (PK), and the internal ribosome entry site (IRES) [7]. The single ORF is translated into a single polyprotein which is eventually processed into four structural proteins VP4 (1A), VP2 (1B), VP3 (1C), VP1 (1D), and eight non-structural proteins (L^pro^, 2A, 2B, 2C, 3A, 3B, 3C^pro^, 3D^pol^) [1,5,7].

VP1, VP2, and VP3 are exposed on the capsid surface while VP4 is embedded inside the capsid. VP1 plays an important role in serotype-specificity, host cell attachment, and antigenicity. Hence, VP1 is widely used for the molecular characterization and epidemiological analysis of FMDV strains. It is the most variable region among the capsid proteins [5,7]. Mutations in VP1 are considered crucial as three major antigenic sites (sites 1, 3, and 5) are located within VP1 [5,8].

FMDV has evolved into seven serologically distinct types (Euro-Asian serotypes O, A, Asia 1, C; Southern African Territories serotypes SAT-1, SAT-2, SAT-3), each of which is subdivided into diverse genetic variants [1,5] causing FMD epidemics in different countries each year.

In Bangladesh, FMD outbreaks due to three FMDV serotypes O, A, and Asia 1 were reported regularly since 2009, with serotype O being responsible for 85% of the outbreaks [9]. Among the 11 topotypes of O, only the Middle East-South Asia (ME-SA) topotype is found in Bangladesh. Within ME-SA topotype, several lineages emerged in circulation of which Ind-2001, PanAsia, and PanAsia-2 are the most dominant [[10], [11], [12]]. Other endemic lineages that were reported under the ME-SA topotype such as Ind-2011, Pak-98 and Srl-97 were mostly confined to specific areas and did not exhibited widespread transmission [[13], [14], [15]]. Ind-2001 lineage has five sublineages, Ind-2001a, b, c, d and e [12]. In Bangladesh, during 2012–13, Ind-2001d sublineage of serotype O was predominant and during the same timespan two novel sublineages, Ind-2001BD1 (or Ind-2001e) and Ind-2001BD2 were detected [3]. Ind-2001BD1 was named as Ind-2001e by the World Reference Laboratory for Foot-and-Mouth Disease, WRLFMD [16]. In recent years, Ind-2001e (Ind-2001BD1) became the dominant sublineage in circulation replacing the Ind-2001d sublineage in Bangladesh [17]. This pattern of FMDV Ind-2001 lineage circulation was also observed in the Indian subcontinent [12]. A new lineage called SA-2018 of ME-SA topotype under serotype O evolved recently and was first reported in India in 2018 [18]. This lineage was also detected to circulate in Bangladesh by our laboratory, Microbial Genetics and Bioinformatics Laboratory (MGBL), Department of Microbiology, University of Dhaka, in 2021 but as a distinct sublineage, named MYMBD21 [17] and in this study, we presented the first complete genome sequence of an isolate, BAN/MY/My-466/2021 (My-466) (accession no. OP957418.1) [19] that belong to the MYMBD21 sublineage under the SA-2018 lineage. This study included comparative analysis at sequence level to decipher which mutations were involved in the emergence of a new genetic lineage from previously established lineage. Also, emergence of any new genetic variant raises a possibility of vaccine escape, therefore, we presented sequence variations from available vaccine strains and homology modelling was also added to demonstrate the association of the sequence heterogeneity with the structural variation at antigenic sites of capsid proteins. This study was limited to the reporting of variations at the sequence level but not in the antigenic level. Characterization of the full genome of this emerging strain would provide necessary data required to track its transmission pattern, assess the efficacy of existing vaccines against the subtype, and take proper steps to prevent future outbreaks caused by the newly emerged lineage.

## 2 Materials and methods

### 2.1 Ethical declarations

The approval for sample collection was taken from the Animal Experimentation Ethical Review Committee (AEERC), Faculty of Biological Sciences, University of Dhaka **(Ref:66/Biol. Sci./2018**–**19; Date: 14**–**11**–**2018)** (Supplementary Fig. S1). All methods were performed following the guidelines approved by the AEERC.

### 2.2 Sample collection and processing

During 2019–2021, 24 clinical FMD outbreaks were reported in nine different districts of Bangladesh of which 22 outbreaks were due to serotype O and only 2 were caused by serotype A. The serotype of the isolates was confirmed by the VP1 sequence data analysis. All the isolates collected from the Mymensingh district in 2021 were of the novel lineage O/ME-SA/SA-2018 [17]. In this study, we presented the whole genome sequence analysis of one of the isolates collected from the Mymensingh district that was designated as BAN/MY/My-466/2021 (My-466). The tongue epithelium tissue sample was collected on December 30, 2021, from FMD-infected cattle at a dairy farm in Mymensingh district, Bangladesh. Sample collection was done by a registered veterinarian following proper ethical guidelines. The sample was transported immediately to the laboratory at 4 °C temperature after collection and stored at −80 °C before virus isolation and RNA extraction.

### 2.3 RNA extraction and cDNA synthesis

Viral RNA extraction was done by using Maxwell® 16 LEV RNA Cartridge in an automated Maxwell® nucleic acid extraction instrument following the manufacturer's protocol (Promega, USA). The extracted RNA was reverse transcribed into complementary DNA (cDNA) using the ImProm-II™ Reverse Transcription System (Promega, USA) as per the manufacturer's instruction.

### 2.4 Complete genome amplification and sequencing

Polymerase Chain Reaction (PCR) amplification method using 17 sets of primer pairs [[20], [21], [22], [23], [24], [25], [26]] spanning the entire genome of the isolated virus was employed to amplify 17 overlapping fragments of the virus genome. Primers used in amplification are listed in Supplementary Table S1. PCR amplicons were purified using the Wizard® SV Gel and PCR Clean-Up System (Promega, USA). Purified amplicons were sequenced from Macrogen, Inc. Seoul, South Korea.

### 2.5 Complete genome assembly and annotation

Overlapping PCR amplicons of the entire genome of the isolate, My-466 were assembled into a complete consensus sequence using SeqMan version 7.0.0 (Lasergene, DNASTAR, USA). All the parameters of the assembly project were set as default. Degenerate traces shown in the consensus were fixed by subsequent National Centre for Biotechnology Information Basic Local Alignment Search Tool (NCBI BLAST) searches [27]. The serotype identification was performed by NCBI BLAST search and subsequent genome annotation was performed by comparison with the NCBI Ref Seq (Reference sequence for Foot-and-Mouth Disease Virus-accession no. AF308157.1). Pairwise and multiple alignments of Ref Seq and the complete genome of My-466 were performed in ClustalW [28] of MEGA11 [29] to annotate the complete genome.

The complete genome sequence of My-466 was submitted to the NCBI GenBank database (https://www.ncbi.nlm.nih.gov) under accession no. OP957418.1 [19].

### 2.6 Phylogenetic analysis

For phylogenetic analysis of the complete genome of My-466, a total of 20 complete genomes of FMDV reference sequences were retrieved from the GenBank database of NCBI (Supplementary Table S2). A total of 85 sequences were taken into consideration for VP1-based phylogeny for determining the lineage of the isolated virus (My-466) (Supplementary Table S3). Multiple sequence alignment was performed in the ClustalW program of MEGA 11 software [29] and the tree was constructed using the Neighbor-Joining method [30] based on the Kimura-2 parameter model [31]. A discrete Gamma distribution of a value of 1 was used to model evolutionary rate differences among sites and for the evaluation of the reliability of the branching point bootstrap value of 1000 was used. Alignment gaps, missing data, and ambiguous bases of about fewer than 5% were allowed at any position as all the sequences were not completely aligned on the full range.

### 2.7 Comparative sequence analysis

Nucleotide and amino acid sequence identity of My-466 against other reference strain (BHU_27/2004, accession no. HQ268524.1), field vaccine strain (O/India/R2/75, accession no. AF204276.1) [32] and proposed local vaccine strain (BAN/TA/Dh-301/2016, accession no. MK088170.1) [33] was calculated using a global alignment tool based on the Needleman-Wunsch algorithm [34] in NCBI. Genetic distance between groups was calculated in MEGA11 [29] using the Kimura-2 parameter method [31].

### 2.8 Protein variability analysis

Protein variability of capsid proteins based on the Wu-Kabat method [35] was calculated in Protein Variability Server [36] using FASTA protein alignment of capsid proteins of My-466 and vaccine strains as input where My-466 capsid proteins were selected as the reference.

### 2.9 Prediction of the three-dimensional (3D) structure of capsid proteins

Homology modeling of capsid proteins comprising the antigenic region (VP1, VP2, VP3) of the isolate My-466 and vaccine strains was performed using the SWISS-MODEL server [37,38]. The quality of three-dimensional structures was validated using the Ramachandran plot [39] analysis in *Molprobityv4.4* [40]. The 3D models were visualized using PyMOL [41] software.

## 3 Results

### 3.1 Complete genome annotation

Annotations for the complete genome of BAN/MY/My-466/2021 or My-466 (NCBI: OP957418.1) are listed in Supplementary Table S4.

The complete genome of My-466 (NCBI: OP957418.1) is 8216 nucleotides (nt) in length with 6996 nt open reading frame (ORF) which encodes a 2332 amino acids long polyprotein. ORF is flanked by an 1100 nt 5ꞌ untranslated region (5ꞌ UTR) and approximately 120 nt 3ꞌ UTR. In the sequence, A, T(U), G, and C are 24.8%, 21.3%, 25.8%, and 28.2%, respectively. The 5ꞌ UTR contains S-fragment (1–370 nt), poly (C) tract (371–386 nt), Pseudoknots (387–543 nt), and Internal Ribosome Entry Site (IRES) (544–1100 nt). The 3ꞌ UTR contains poly (A) tail. The structural part is 2208 nt long encoding structural proteins of a total of 736 amino acids and the non-structural part (L^pro^, 2A, 2B, 2C, 3A, 3B, 3C, 3D) consists of 4,788 nt encoding 1596 amino acids.

### 3.2 Complete genome analysis

#### 3.2.1 Local alignment of the complete genome

In the Basic Local Alignment Search Tool (BLAST) search, the closest hit of My-466 complete genome was FMDV type O isolate BHU_27/2004, complete genome (accession no. HQ268524.1) with 92.85% identity covering 99% of the query sequence, whereas the max score of the hit against query was 11900 and Expect value (E-value) of getting another hit in the database was 0. This isolate belonged to the PanAsia-2 lineage. The next closest hit was the complete genome of TUR/12/2013 (accession no. KM268895.1) having 100% query coverage and 92.59% identity. About 92.44% identity was found for another hit, MAY/1/2004 (accession no. HQ632770.1). The immediate next hits were FMDV type O isolates BAN/BO/Na-161/2013 (accession no. MK071699.1), Tibet/CHA/99 (accession no. AJ539138.1), BAN/GO/Ka-236(Pig)/2015 (accession no. KX712091.1), TUR/18/2010 (accession no. JX040491.1) and PAK/14/2017 (accession no. MH784405.1) sharing about 92% identity (Supplementary Fig. S2). Any complete genome for O/ME-SA/SA-2018 lineage was not found in the BLAST search.

#### 3.2.2 Phylogenetic analysis

**Fig. 1:**
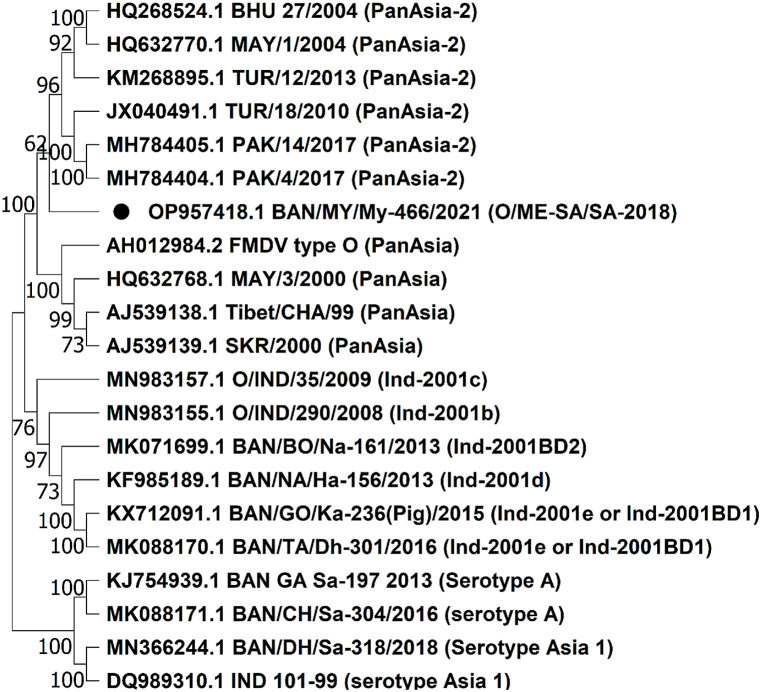
**Complete genome-based phylogenetic reconstruction.** The tree was constructed based on the Neighbor-Joining method using the Kimura-2 parameter model in MEGA11. The tree showed the formation of a distinct branch by the O/ME-SA/SA-2018 lineage (BAN/MY/My-466/2021 isolate) from the PanAsia-2 lineage. The isolate under this study was labeled with a bullet shape. Serotype A and Asia 1 isolates were taken as outgroups.

Complete genome-based phylogeny (Fig. 1) revealed the formation of a distinct clade by the isolate My-466 of O/ME-SA/SA-2018 lineage from other established lineages (Ind-2001, PanAsia, PanAsia-2) of ME-SA topotype under serotype O which indicated that this lineage is a novel one with no previous reporting of the complete genome of this lineage.

**Fig. 2:**
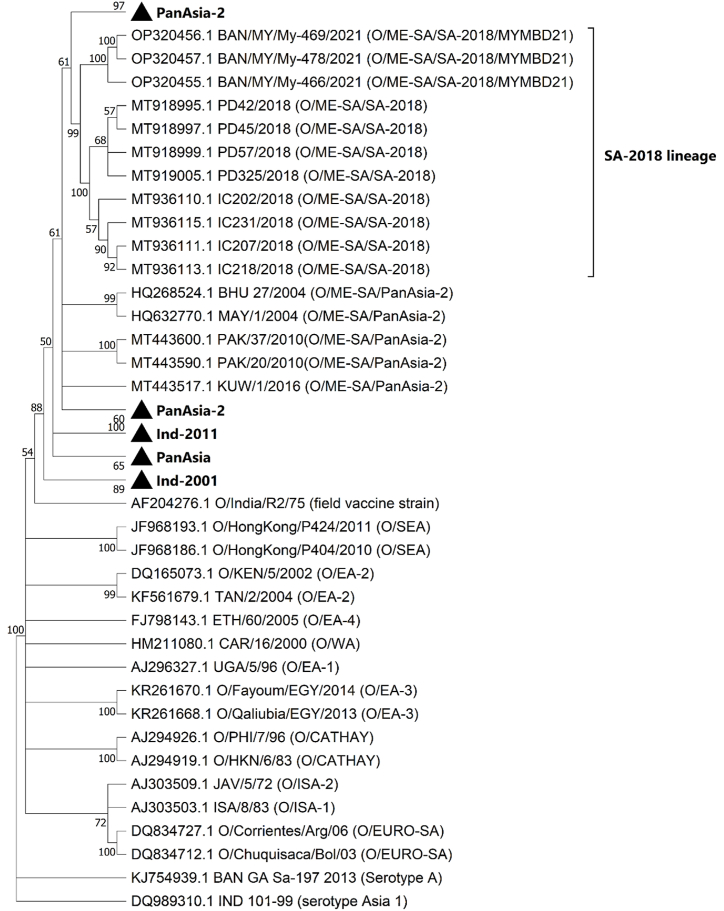
**VP1 based phylogenetic reconstruction.** The tree was constructed based on the Neighbor-Joining method using the Kimura-2 parameter model in MEGA11. The tree showed clustering of MYMBD21 isolates (OP320455.1-OP320457.1) forming a distinct clade with other isolates of O/ME-SA/SA-2018 lineage. This lineage showed possible evolution from PanAsia-2 lineage.

In a previous study carried out by our laboratory, detailed analyses on the VP1 sequence of the isolate provided evidence of the circulation of the SA-2018 lineage in Bangladesh with a mutational trend of emerging novel sublineage, MYMBD21 [17]. In this study, VP1-based phylogeny (Fig. 2) and evolutionary divergence of 6% between the Indian isolates of SA-2018 lineage and the MYMBD21 isolate, My-466 calculated in MEGA11 (Supplementary Fig. S3) also confirmed the existence of My-466 as MYMBD21 sublineage under the SA-2018 lineage. In Fig. 1, the isolate showed a possible evolution of the SA-2018 lineage from the PanAsia-2 lineage.

#### 3.2.3 Sequence comparison of BAN/MY/My-466/2021 against the reference sequence

For comparative complete genome analysis, an isolate, BHU_27/2004 (accession no. HQ268524.1) under PanAsia-2 lineage was considered as a reference sequence because BHU_27/2004 exhibited the highest identity with the isolate in BLAST search and no complete genome for the O/ME-SA/SA-2018 lineage was available. The nucleotide and protein identity of My-466 sequences against reference strain (BHU_27/2004) is presented in Table 1.

**Table 1:** Percentage of nucleotide and protein identity between BAN/MY/My-466/2021 and reference sequence (BHU_27/2004).

Gene Segments	Nucleotide Identity (%)	Protein Identity (%)
5ꞌ UTR	91%	–
Lpro	89%	95%
VP4	96%	99%
VP2	92%	99%
VP3	94%	97%
VP1	92%	98%
2A	92%	100%
2B	95%	97%
2C	94%	99%
3A	91%	98%
3B	92%	100%
3C	93%	99%
3D	94%	99%
3ꞌ UTR	∼83%	–

The 5ꞌ UTR of My-466 demonstrated 91% nucleotide identity with 95 substitutions including 12 gaps (Supplementary Fig. S4). Within 5ꞌ UTR, different types of mutations (insertion, deletion, and substitution) were detected in the S-fragment, poly (C) tract, Pseudoknot and IRES region of My-466 sharing 91%, 44%, 89%, and 94% nucleotide identity with the reference strain, respectively (Supplementary Figs. S5, S6, S7, and S8). The S-fragment of 5ꞌ UTR was found to be 91% identical to the reference sequence with 30 nucleotide changes, 2 insertions (279th, 358th), and 1 deletion (147th position) (Supplementary Fig. S5). In the poly (C) tract, 7 cytosines (C) insertion occurred (Supplementary Fig. S6). Pseudoknot region was 89% (139/157) identical and 1 adenine insertion at 50th position was detected (Supplementary Fig. S7). Internal Ribosome Entry Site (IRES) region was 6% divergent and deletion of only one cytosine was found at position 536 (Supplementary Fig. S8). The 3ꞌ UTR portion was detected as highly variable sharing 83% nucleotide identity. Insertion of two nucleotide was detected and twelve nucleotide substitutions were found. The full length of the poly (A) tail could not be determined by the sequencing method used, however, 7 extra adenines compared to the reference strain were detected in our sequence (Supplementary Fig. S9). Among the structural part (VP4-VP1), VP2 and VP1 showed more nucleotide divergence (8%) than VP3 and VP4. The VP4 nucleotide (96%) was more conserved than other coding sequences showing only 1 amino acid change in the encoded protein compared to the reference (Table 1; Supplementary Fig. S10). Among the non-structural regions (Lpro, 2A, 2B, 2C, 3A, 3B, 3C, 3D), Lpro and 3A regions showed more nucleotide variations with only 89% and 91% nucleotide identity, respectively while 100% conserved protein were encoded by the 2A and 3B regions (Table 1; Supplementary Figs. S11 and S12). Based on the amino acid sequence analysis, fully conserved regions (100%) against the reference were 2A, and 3B whereas VP4, VP2, 2C, 3C, and 3D regions were 99% conserved. About 2% amino acid difference was observed in the VP1 as well as in the 3A region and about 3% amino acid variation was detected in the VP3 and 2B encoded proteins (Table 1). The highest variation (5%) was detected in the Lpro region where 10 amino acid substitutions occurred (Supplementary Fig. S11).

#### 3.2.4 Mutational analysis against vaccine strains

Mutational analysis of My-466 capsid protein was performed against the current field vaccine strain, O/India/R2/75 (accession no. AF204276.1) [32] and the proposed local vaccine strain, BAN/TA/DH-301/2016 (accession no. MK088170.1) [33].

Capsid proteins were 4% (30 substitutions out of 736 amino acid residues) diverged from the field vaccine strain (Supplementary Fig. S13) and 3% divergence (23 substitutions out of 736 amino acid residue) was observed from the proposed local vaccine strain (Supplementary Fig. S14). Amino acid changes in each of the four capsid proteins (VP4, VP2. VP3, and VP1) of My-466 against field vaccine strain are listed in Table 2. Against the field vaccine strain, VP4 was found to be 99% conserved with only one mutation, I80F. About 97% (212/218) VP2 homology was found against the field vaccine strain in which S70P, A74V and H79Y mutations were detected in the antigenic region, B–C loop. The mutation, S70P demonstrated polar serine (S) conversion into nonpolar proline (P) and H79Y showed a substitution of positively charged histidine (H) to uncharged tyrosine (Y). Two mutations, Q133T and N134K were detected in the E-F loop (132–135 or 131–134) of VP2 of which uncharged asparagine (N) was substituted by positively charged lysine (K). VP3 demonstrated a 5% divergence from the field vaccine strain exhibiting 12 amino acid changes. In the B–B knob region of the VP3, only one amino acid substitution (G60D) occurred at the 60th amino acid residue. VP1 of the field vaccine strain and My-466 shared 95% homology with 11 mismatches in amino acid sequences of which 6 of the mutations (D138E, G139S, S140H, V141A, N143S, I144V) were in the G-H loop. G139S showed changes in polarity. At the 140th position, uncharged serine (S) was changed into positively charged histidine (H). No mutations occurred in the B–C loop (43–59) and only asparagine (N) to serine (S) conversion at 197th position was observed in the C-terminus (190–213) (Table 2).

**Table 2:** Amino acid changes in BAN/MY/My-466/2021 against field vaccine strain (O/India/R2/75).

Capsid Proteins (length)	Protein Identity (%)	Regions (amino acid positions)	Amino acid position in each protein	Field vaccine strain (O/India/R2/75)	BAN/MY/My-466/2021
VP4 (85 amino acid residues)	99%		80	I	F
VP2 (218 amino acid residues)	97%	B–C loop (70–80) [42,43]	70	S	P
			74	A	V
			79	H	Y
		E-F loop (131–134 or 132–135) [42,43]	133	Q	T
			134	N	K
			154	V	M
VP3 (220 amino acid residues)	95%		8	S	G
			14	L	F
			25	V	A
			44	F	L
			56	R	H
		B–B knob (58–61) [42,44]	60	G	D
			86	I	M
			96	H	Q
			195	E	D
			215	V	I
			219	R	T
			220	D	E
VP1 (213 amino acid residues)	95%		82	Y	H
			96	N	K
			123	Q	H
			126	L	M
		G-H loop (130–160) [3,45]	138	D	E
			139	G	S
			140	S	H
			141	V	A
			143	N	S
			144	I	V
		C-terminal (190–213) [3,46]	197	N	S

Sequence Identity and amino acid substitutions of capsid proteins between My-466 and the proposed local vaccine strain (BAN/TA/Dh-301/2016) are listed in Table 3. VP4 of My-466 was fully conserved against the VP4 of the BAN/TA/Dh-301/2016. The VP2 was 98% identical to the local vaccine strain showing 5 amino acid changes demonstrating only H79Y (positive histidine to aromatic tyrosine) mutation occurred in the B–C loop and Q133T in the E-F loop that were also detected against the field strain. The VP3 was 97% homologous (213/220) to the VP3 of the local vaccine strain but no mutation in the B–B knob was found. Comparison of BAN/MY/My-466/2021 VP1 with the proposed local vaccine strain also revealed 95% identity with 11 mutations in VP1, of which 5 were found in the G-H loop region (K138E, G139S, A140H, V141A, N143S). The positive to negative charge conversion was detected (K138E) at position 138 and at the 140th position, uncharged alanine (A) was replaced by positively charged histidine (H). Changes in polarity occurred in the case of G139S in the G-H loop as well as in the B–C loop (I43T). Two mutations (E197S and Q198E) were observed in the C-terminal region in which negatively charged glutamate (E) transformed into uncharged serine (S) at 197th residue and uncharged glutamine (Q) was replaced by negatively charged glutamate (E) at residue 198 (Table 3).

**Table 3:** Amino acid changes in BAN/MY/My-466/2021 against proposed local vaccine strain (BAN/TA/Dh-301/2016).

Capsid Proteins (length)	Protein Identity (%)	Regions (amino acid positions)	Amino acid position in each protein	Proposed Local vaccine strain (BAN/TA/Dh-301/2016)	BAN/MY/My-466/2021
VP4 (85 amino acid residues)	100%	–	–	–	–
VP2 (218 amino acid residues)	98%		23	I	T
		B–C loop (70–80) [42,43]	79	H	Y
			93	G	S
		E-F loop (131–134 or 132–135) [42,43]	133	Q	T
			191	N	T
				
VP3 (220 amino acid residues)	97%		8	S	G
			14	L	F
			44	F	L
			131	K	E
			174	A	T
			215	V	I
			220	Q	E
VP1 (213 amino acid residues)	95%		13	T	A
		B–C loop (43–59) [3,47]	43	I	T
			96	A	K
			126	L	M
		G-H loop (130–160) [3,45]	138	K	E
			139	G	S
			140	A	H
			141	V	A
			143	N	S
		C-terminal (190–213) [3,46]	197	E	S
			198	Q	E

**Fig. 3:**
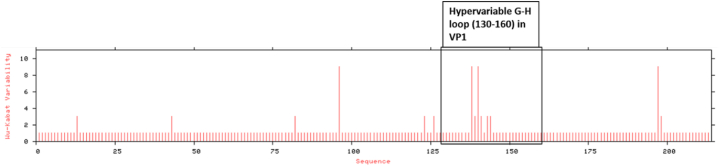
Wu-Kabat protein variability plot of VP1 of BAN/MY/My-466/2021 against the field and the proposed local vaccine strains.

Amino acid variability calculation using Wu-Kabat protein variability co-efficient also confirmed that VP1 is the most variable region and VP4 is the most conserved region among the capsid proteins (Supplementary Fig. S15). Within the VP1 region, the most variability was found in amino acid sequence between positions 138–144 which is G-H loop (Fig. 3).

#### 3.2.5 Three-dimensional (3D) modelling of capsid proteins

**Fig. 4:**
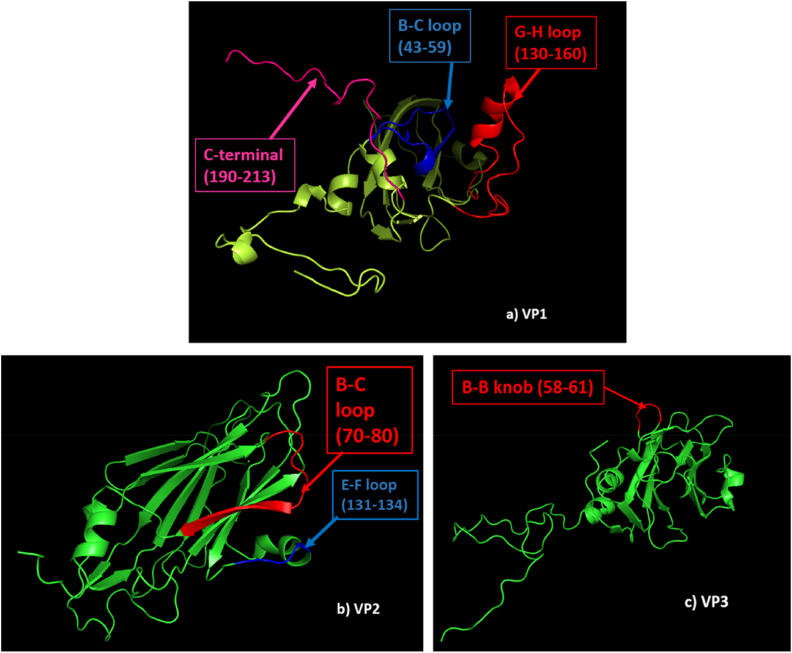
**Three-dimensional model of capsid proteins.** In 4a, 4b and 4c, 3D models of VP1, VP2, and VP3 of BAN/MY/My-466/2021 are illustrated indicating their major antigenic regions, respectively. (a) VP1 model: B–C loop (blue), G-H loop (red) and C-terminal region (pink); (b) VP2 model: B–C loop (red), E-F loop (blue); (c) VP3 model: B–B knob (red).

FMDV surface proteins, VP1, VP2 and VP3 possess major antigenic regions of the virus which are indicated in 3D models of the capsid proteins in Fig. 4a, b, 4c. Superimposed capsid protein model against vaccine strains were also generated to detect whether any structural change is associated with mutations detected in major antigenic sites. The Ramachandran plot for all structures showed >92% of the residues within the favored region. The result of the quality assessment of models can be found in Supplementary Figs. S16–S24.

**Fig. 5:**
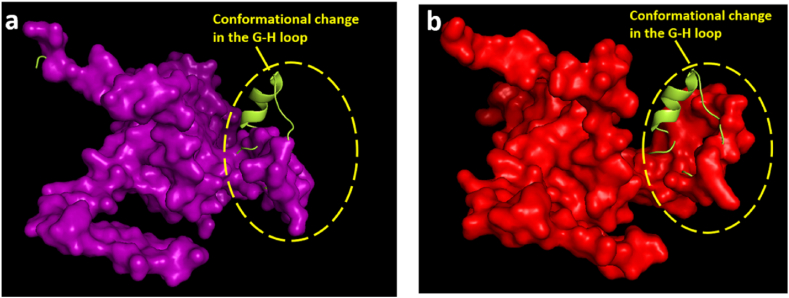
**Three-dimensional model of VP1.** VP1 of BAN/MY/My-466/2021 was presented in cartoon style (yellow), VP1 of available field vaccine strain (O/India/R2/75) (purple) and proposed local vaccine strain (BAN/TA/Dh-301/2016) (red) was presented in surface style. (a) VP1 of BAN/MY/My-466/2021 superimposed on VP1 of available field vaccine strain (O/India/R2/75); (b) VP1 of BAN/MY/My-466/2021 superimposed on VP1 of proposed local vaccine strain (BAN/TA/Dh-301/2016). (For interpretation of the references to colour in this figure legend, the reader is referred to the Web version of this article.)

In Fig. 5, a major conformational displacement in the G-H loop was observed when the VP1 model of My-466 was superimposed on both VP1 models of field (O/India/R2/75) and proposed local vaccine (BAN/TA/Dh-301/2016) strains which indicated structural heterogeneity of the crucial antigenic site, G-H loop in My-466 against both vaccine strains. No conformational change was observed in the superimposition of VP2 and VP3 models against both vaccine strains (Supplementary Fig. S25, Supplementary Fig. S26).

## 4 Discussion

BAN/MY/My-466/2021 or My-466 isolate belongs to a novel sublineage, MYMBD21 under SA-2018 lineage which was confirmed in a previous study carried out by our laboratory and also in the VP1-based phylogeny in this study (Fig. 2). The O/ME-SA/SA-2018 lineage was first detected in India in 2018 [18] and later intruded in Bangladesh in 2021 as novel MYMBD21 sublineage [17]. Complete genome information of this new lineage was not available before this study. From the phylogenetic analysis (Fig. 1; Fig. 2) and NCBI BLAST analysis, it can be demonstrated that SA-2018 isolates are more closely related to the PanAsia-2 isolates exhibiting possible emergence from the PanAsia-2 lineage. Therefore, this study presented a genome-wide comparative analysis of the novel genetic variant MYMBD21 of the SA-2018 lineage against the PanAsia-2 lineage (Reference sequence: BHU_27/2004) as well as with the available vaccine strains (O/India/R2/75; BAN/TA/Dh-301/2016) to detect any major sequence variations affecting the target epitope of the vaccine.

Comparative analysis demonstrated that the complete genome of My-466 (SA-2018 lineage) showed 92.85% identity with reference sequence, BHU_27/2004 (PanAsia-2 lineage). My-466 shared 96% (706/736) capsid protein (VP4-VP1) homology with the capsid region of the field vaccine strain, O/India/R2/75, and 97% (713/736) homology with the proposed local vaccine strain, BAN/TA/Dh-301/2016 (Supplementary Figs. S13 and S14). Among the mutations observed in VP2 of My-466 against both the vaccine strains, H79Y mutation could be associated with extended receptor tropism as substitutions between the amino acid position 78–80 of VP2 might alter the orientation of the G-H loop of VP1 [5,48]. Amino acid exchanges at positions 133rd and 134th (Q133T, N134K) could be considered crucial for receptor binding as these positions are located in the αB helix of VP2 (residues 133–138) which forms the part of depression used for heparan sulfate (HS) binding by the virus [5,49,50]. In VP3, one mutation (G60D) in the antigenic region, B–B knob (58–61) was found against the field vaccine strain where uncharged glycine (G) was converted into negatively charged aspartate (D). B–B knob forms one of the walls of the depression of HS binding [5,51] and mutations at this site can affect the antigenicity of the virus.

The amino acid sequence-based comparison revealed that VP1 was 95% homologous to both field and local vaccine strains. Mutations at amino acid positions, 138, 139, 140, 141, and 143 of the G-H loop and at the 197th amino acid of the C-terminus occurred in My-466 VP1 in comparison to both the vaccine strains (Table 2; Table 3). In BAN/MY/My-466/2021, valine (V) instead of isoleucine (I) was found at the 144th position of VP1 amino acid sequences against the field vaccine strain which is a crucial mutation as 144th, 148th, 154th, and 208th amino acid positions are considered critical for the formation of antigenic site 1 in VP1 [52]. Frequent mutations at residues, 138, 139, 142, 143, and 144 were also observed in other studies [43,49,53,54]. In the C-terminal region, changes at residues 197 and 198 were detected that were reported in prior studies as infrequent mutation sites compared to the other positions [5,[53], [54], [55]].

While VP1 was found to be the most variable region, VP4 was observed to be the most conserved region compared to other capsid proteins (Supplementary Fig. S15). Among the antigenic regions, the majority of the amino acid substitutions were detected in the G-H loop of VP1 against both vaccine strains. The G-H loop was found to be the most variable region of VP1 carrying 5–6 mutations at critical amino acid positions (Table 2; Table 3). Other studies also reported high variability in the hypervariable region of the G–H loop between amino acid positions 130–160 (for type O) which contributes to the major antigenic sites on the capsid coding region [5,46]. These alterations in the receptor binding sites of the capsid could facilitate the bypass of host response elicited by vaccine strains and the confirmation of this possibility requires further serological analysis.

To know whether these mutations were related to structural changes in VP1, superimposition of the three-dimensional (3D) structures was performed. In the case of VP2 and VP3, no conformational change in My-466 against vaccine strains was observed in the superimposed 3D model which indicated that the amino acid variations in those regions did not contribute to significant structural variation in antigenic sites (Supplementary Figs. S25 and S26). Superimposition of the 3D model of VP1 of My-466 with that of both vaccine strains showed that the G-H loop of the isolate did not align with the G-H loops of vaccine strains (Fig. 5). This delineated that amino acid substitutions were related to the significant structural variations of the epitope region, the G-H loop of My-466 with that of the vaccine strains. Antigenic site 1 contains a highly conserved Arg-Gly-Asp or RGD motif (145–147) which is located at the apex of the G-H loop and interacts with host receptors, the integrin, to induce virus entry [5,6,56]. Variable amino acids around this motif might lead to changes in the immune specificity and the generation of newer variants. Substitutions in critical sites (S140H, A140H, V141A, N143S, I144V) adjacent to the conserved RGD motif (145–147) were detected (Table 2; Table 3) which might be responsible for the conformational changes in the antigenic sites. To predict antigenic heterogeneity or to assess the efficacy of existing vaccine strains, serological tests such as in-vitro 2-dimensional microneutralization test should be carried out.

Coexistence of different strains of FMDV in a defined geographical region without cross-protective immunity leads to devastating impacts during outbreaks. The best possible way to counter the adversity is prevention through rapid diagnosis, the fastest acknowledgement of newer strains in circulation, vaccination to avoid its outbreak or prevalence and other steps. Genome-wide analyses of novel FMDV variants contribute to deciphering the underlying mutations responsible for their emergence and provide the necessary knowledge to check whether the existing control measures could contain the spread of the new variant or not. Molecular understanding of novel strains provides prerequisite knowledge to adopt more appropriate containment strategies so that more devastating disease outbreaks by new genetic variants can be prevented before they go beyond control.

## 5 Conclusions

The complete genome information of the emerging novel FMDV variant is crucial for understanding its evolutionary history, mutational frequency and the transmission pattern. Genome-wide analyses of novel variant SA-2018/MYMBD21 yield newer information and add valuable data to our prior knowledge to track the mutational pattern of the emerging novel variant and thus facilitate faster detection of any significant changes in antigenicity that could compromise the efficacy of the existing vaccines. Therefore, complete genome information of the emerging lineage, O/ME-SA/SA-2018 would be valuable to keep track of its circulation and to prevent, predict, or at least warn of near future outbreaks by this strain.

## Ethics statement

The approval for sample collection was taken from the Animal Experimentation Ethical Review Committee (AEERC), Faculty of Biological Sciences, University of Dhaka **(Ref:66/Biol. Sci./2018**–**19; Date: 14**–**11**–**2018)** (Supplementary Fig. S1). All methods were performed following the guidelines approved by the AEERC.

## Funding

This research received no external funding.

## Institutional review board statement

The study protocol was approved by the Animal Experimentation Ethical Review Committee (AEERC), Faculty of Biological Sciences, University of Dhaka (Ref:66/Biol. Sci./2018–19; Date: 14-11-2018).

## Informed consent statement

Not applicable.

## Data availability statement

The complete genome sequence of BAN/MY/My-466/2021 (NCBI: OP957418.1) has been published in NCBI GenBank (https://www.ncbi.nlm.nih.gov) under the accession number OP957418.1. Supplementary materials supporting the results of the study are available in this article as supplementary Data.

## CRediT authorship contribution statement

**Humaira Anjume:** Writing – original draft, Investigation, Formal analysis, Conceptualization. **Kazi Alamgir Hossain:** Writing – review & editing, Investigation, Conceptualization. **Anamica Hossain:** Writing – original draft. **M. Anwar Hossain:** Writing – review & editing, Supervision, Conceptualization. **Munawar Sultana:** Writing – review & editing, Supervision, Conceptualization.

## Declaration of competing interest

The authors declare that they have no known competing financial interests or personal relationships that could have appeared to influence the work reported in this paper.
