# Supplementary material for: Complete genome characterization of foot-and-mouth disease virus My-466 belonging to the novel lineage O/ME-SA/SA-2018
Source: Heliyon. 2024 Feb 20;10(5):e26716. doi: 10.1016/j.heliyon.2024.e26716 (PMC11713259; doi:10.1016/j.heliyon.2024.e26716)
Supplement: Multimedia component 1 [file mmc1.docx]

**Supplementary Data**

**Supplementary** **Table S1 Primer list for complete genome amplification**

| **Primer**  **Name** | **Sequence (5ꞌ-3ꞌ)** | **Location** | **Position** | **Ta**  **(**$\boldsymbol{℃}$**)** | **Reference** |
| --- | --- | --- | --- | --- | --- |
| **20F** | TTGAAAGGGGGCRCTAGGGT | **5’UTR** | 1-20 | 55 | (Sultana *et al.* 2014) |
| **M1R** | CGGTAAAACTTGGGGGGGGGGGGGGGGTGAAAGGC | **5’UTR** | 360-392 | 60 | (Ma *et al.* 2014) |
| **LF1F^a^** | CCCCCCTAAGTTTTACCGTCGTTCCCG | **5’UTR** | 378-405 | 60 | (Sanyal *et al.* 2004) |
| **P2R** | ACATGATGGGTCCGTTAGGA | **5’UTR** | 843-862 | 55 | (Ali *et al.* 2016) |
| **1F** | GCCTGGTCTTTCCAGGTCT | **5’UTR** | 640– 658 | 55 | (Reid *et al.* 2000) |
| **1OEXR** | CCCTCGTGYAGYTCAAGACC | **VP4** | 1329–1348 | 55 | (Sultana *et al.* 2014) |
| **2OEXF** | GGTCTTGARCTRCACGAGGG | **VP4** | 1329 – 1348 | 55 |  |
| **2OEXR** | CCGTAGACRCCTTTGTGGTC | **VP2** | 2172 – 2191 | 55 |  |
| **3OEXF** | GACCACAAAGGYGTCTACGG | **VP2** | 2172 – 2191 | 55 |  |
| **3OR** | AAGTGCAGGTTRATGGTGCC | **VP3** | 2877– 2896 | 55 |  |
| **4OF** | CAAGGTSTATGCCAACATCG | **VP3** | 2507– 2526 | 55 |  |
| **4OR** | RTYTGCATCAGGTCCAACAC | **VP1** | 3378– 3397 | 55 |  |
| **5OEXF** | GAGAACTACGGTGGTGAGAC | **VP1** | 3276– 3295 | 55 |  |
| **NK61** | GACATGTCCTCCTGCATCTG | **2B** | 3971 –3994 | 55 | (Samuel and Knowles 2001) |
| **NSP 1F** | GAGACGTYGAGTCCAACCC | **2B** | 3939-3958 | 55 | (Abdul-Hamid *et al.* 2011) |
| **NSP 1R** | CTTCTGAGGCGATCCATG | **2C** | 4517-4535 | 55 |  |
| **NSP 2F** | CAGCTCARAGCACGTGACAT | **2C** | 4423-4443 | 55 |  |
| **NSP 2R** | GCCATRGGCGGGATRAA | **2C** | 4972-4989 | 55 |  |
| **NSP 3F** | TGACCACTTYGACGGTTA | **2C** | 4860-4878 | 55 |  |
| **NSP 3R** | ACCATCCCCTCRAAGAAYTC | **3A** | 5449-5469 | 55 |  |
| **NSP 4F** | CGRAGGTTYCACTTTGAC | **3A** | 5098-5116 | 55 |  |
| **NSP 4R** | CATRATCACTATGTTTGCCA | **3A** | 5585-5605 | 55 |  |
| **NSP 5F** | GAATTCTTTGAGGGGATGGT | **3A** | 5449-5469 | 55 |  |
| **NSP 5R** | CACTTTCAAAGCGACAGG | **3C** | 6007-6025 | 55 |  |
| **NSP 6F** | CRAGCTGAAGGACCCTAC | **3B** | 5831-5849 | 55 |  |
| **NSP 6R** | GGGGGTKCCYTTCTTCAT | **3C** | 6377-6395 | 55 |  |
| **NSP 7F** | GGACAGGACATGCTCTCAG | **3C** | 6283-6302 | 55 |  |
| **NSP 7R** | GGACAGGACATGCTCTCAG | **3D** | 6922-6939 | 55 |  |
| **NSP 8F** | ATGCGCAAAACCAAGCT | **3D** | 6736-6753 | 55 |  |
| **NSP 8R** | AATTTGCGGTCCGTTGT | **3D** | 7307-7324 | 55 |  |
| **NSP 9F** | RACCTTCCTGAAGGACGAR | **3D** | 7170-7189 | 55 |  |
| **NSP 9R** | GTCCAGCTCRACTCCCTC | **3D** | 7660-7678 | 55 |  |
| **NSP 10F** | AACGTGTGGGATGTGGA | **3D** | 7393-7410 | 55 |  |
| **T21R** | CAGGAAACAGCTATGACT | **3’UTR** | 8186-8204 | 55 |  |

**Supplementary Table S2 Sequences used in complete genome phylogeny**

| **Sample ID** | **GenBank Accession No.** | **Sero**  **type** | **Topo**  **type** | **Lineage** | **Sublineage** | **References** |
| --- | --- | --- | --- | --- | --- | --- |
| BAN/TA/Dh-301/2016 | MK088170.1 | O | ME-SA | Ind-2001 | Ind-2001BD1/ In-2001e | <https://doi.org/10.1007/s00705-020-04711-6> |
| BAN/GO/Ka-236(Pig)/2015 | KX712091.1 | O | ME-SA | Ind-2001 | Ind-2001BD1/ In-2001e | <https://doi.org/10.1128/genomeA.01150-16> |
| BAN/BO/Na-161/2013 | MK071699.1 | O | ME-SA | Ind-2001 | Ind-2001BD2 | <https://doi.org/10.1128%2FMRA.00705-19> |
| O/IND/290/2008 | MN983155.1 | O | ME-SA | Ind-2001 | Ind-2001b | <https://doi.org/10.1128%2FMRA.00287-20> |
| O/IND/35/2009 | MN983157.1 | O | ME-SA | Ind-2001 | Ind-2001c | <https://doi.org/10.1128%2FMRA.00287-20> |
| BAN/NA/Ha-156/2013 | KF985189.1 | O | ME-SA | Ind-2001 | Ind-2001d | <https://doi.org/10.1128/genomeA.01253-13> |
| MAY/3/2000 | HQ632768.1 | O | ME-SA | PanAsia |  | <https://doi.org/10.1007/s11262-011-0599-3> |
| Tibet/CHA/99 | AJ539138.1 | O | ME-SA | PanAsia |  | <https://doi.org/10.1099/vir.0.18669-0> |
| FMDV type O | AH012984.2 | O | ME-SA | PanAsia |  | <https://doi.org/10.1023/B:VIRU.0000032789.31134.eb> |
| SKR/2000 | AJ539139.1 | O | ME-SA | PanAsia |  | <https://doi.org/10.1023/B:VIRU.0000032789.31134.eb> |
| BHU 27/2004 | HQ268524.1 | O | ME-SA | PanAsia-2 |  | <https://doi.org/10.1093/molbev/msab172> |
| TUR/12/2013 (PanAsia-2) | KM268895.1 | O | ME-SA | PanAsia-2 |  | <https://doi.org/10.1186/1471-2164-15-828> |
| TUR/18/2010 | JX040491.1 | O | ME-SA | PanAsia-2 |  | <https://doi.org/10.1371/journal.pone.0049650> |
| MAY/1/2004 | HQ632770.1 | O | ME-SA | PanAsia-2 |  | <https://doi.org/10.1007/s11262-011-0599-3> |
| PAK/14/2017 | MH784405.1 | O | ME-SA | PanAsia-2 |  | <https://doi.org/10.1128/mra.01397-18> |
| PAK/4/2017 | MH784404.1 | O | ME-SA | PanAsia-2 |  | <https://doi.org/10.1128/mra.01397-18> |
| BAN GA Sa-197 2013 | KJ754939.1 | A | ASIA | G-VII |  | <https://doi.org/10.1128/genomeA.00506-14> |
| BAN/CH/Sa-304/2016 | MK088171.1 | A | ASIA | G-VII |  | <https://doi.org/10.1128/MRA.00031-19> |
| BAN/DH/Sa-318/2018 | MN366244.1 | Asia 1 |  |  |  | <https://doi.org/10.1101/776518> |
| IND 101-99 | DQ989310.1 | Asia 1 |  |  |  | <https://doi.org/10.1016/j.virusres.2008.04.010> |
| BAN/MY/My-466/2021 complete genome | OP957418.1 | O | ME-SA | SA-2018 | MYMBD21 | This study |

**Supplementary Table S3 Sequences used in VP1-based phylogeny**

| **Sample ID** | **GenBank Accession No.** | **Serotype** | **Topotype** | **Lineage** | **Sublineage** | **References** |
| --- | --- | --- | --- | --- | --- | --- |
| BAN/TA/Dh-299/2016 | KY077627.1 | O | ME-SA | Ind-2001 | Ind-2001BD1 | <https://doi.org/10.1111/tbed.12834> |
| BAN/NO/Be-251/2015 | KY077615.1 | O | ME-SA | Ind-2001 | Ind-2001BD1 | <https://doi.org/10.1111/tbed.12834> |
| BAN/LK/Sa-248/2015 | KY077612.1 | O | ME-SA | Ind-2001 | Ind-2001BD1 | <https://doi.org/10.1111/tbed.12834> |
| BAN/TG/Ba-268/2015 | KY077621.1 | O | ME-SA | Ind-2001 | Ind-2001BD1 | <https://doi.org/10.1111/tbed.12834> |
| BAN/TA/Dh-301/2016 | MK088170.1 | O | ME-SA | Ind-2001 | Ind-2001BD1 | <https://doi.org/10.1111/tbed.12834> |
| BAN/GO/Ka-236(Pig)/2015 | KX712091.1 | O | ME-SA | Ind-2001 | Ind-2001BD1 | <https://doi.org/10.1128/genomeA.01150-16> |
| BAN/BO/Na-162/2013 | KY077601.1 | O | ME-SA | Ind-2001 | Ind-2001BD2 | <https://doi.org/10.1111/tbed.12834> |
| BAN LA Sa-137 2013 | KJ175182.1 | O | ME-SA | Ind-2001 | Ind-2001BD2 | <https://doi.org/10.1111/tbed.12834> |
| O/KUW/3/97 | DQ164904.1 | O | ME-SA | Ind-2001 | Ind-2001a | <https://doi.org/10.3201/eid1112.050908> |
| O/UAE/7/97 | AJ318856.1 | O | ME-SA | Ind-2001 | Ind-2001a | <https://doi.org/10.3201/eid1112.050908> |
| O/IND/290/2008 | MN983155.1 | O | ME-SA | Ind-2001 | Ind-2001b | <https://doi.org/10.1128%2FMRA.00287-20> |
| O/IND187/2012 | KC506545.1 | O | ME-SA | Ind-2001 | Ind-2001b | <https://doi.org/10.1016/j.meegid.2013.04.027> |
| O/IND62/2011 | KC506543.1 | O | ME-SA | Ind-2001 | Ind-2001b | <https://doi.org/10.1016/j.meegid.2013.04.027> |
| UAE/4/2008 | KM921876.1 | O | ME-SA | Ind-2001 | Ind-2001c | <https://doi.org/10.1111/tbed.12299> |
| UAE/9/2009 | KJ606983.1 | O | ME-SA | Ind-2001 | Ind-2001c | <https://doi.org/10.1186/1743-422X-11-136> |
| O/IND/35/2009 | MN983157.1 | O | ME-SA | Ind-2001 | Ind-2001c | <https://doi.org/10.1128%2FMRA.00287-20> |
| BAN LA Du-135 2013 | KJ175181.1 | O | ME-SA | Ind-2001 | Ind-2001d | <https://doi.org/10.1111/tbed.12834> |
| BAN TA Dh-184 2013 | KJ175184.1 | O | ME-SA | Ind-2001 | Ind-2001d | <https://doi.org/10.1111/tbed.12834> |
| BAN/GA/Kk-191/2013 | KY077602.1 | O | ME-SA | Ind-2001 | Ind-2001d | <https://doi.org/10.1111/tbed.12834> |
| BAN/NA/Ha-156/2013 | KF985189.1 | O | ME-SA | Ind-2001 | Ind-2001d | <https://doi.org/10.1111/tbed.12834> |
| BAN/PA/Ch-228/2015 | KY077609.1 | O | ME-SA | Ind-2001 | Ind-2001d | <https://doi.org/10.1111/tbed.12834> |
| O/IND182/2011 | KC506553.1 | O | ME-SA | Ind-2011 |  | <https://doi.org/10.1016/j.meegid.2013.04.027> |
| O/IND91/2012 | KC506549.1 | O | ME-SA | Ind-2011 |  | <https://doi.org/10.1016/j.meegid.2013.04.027> |
| Foot-and-mouth disease virus - type O polyprotein gene | AH012984.2 | O | ME-SA | PanAsia |  | <https://doi.org/10.1099/vir.0.18669-0> |
| Tibet/CHA/99 | AJ539138.1 | O | ME-SA | PanAsia |  | <https://doi.org/10.1023/B:VIRU.0000032789.31134.eb> |
| O/NEP/2/99 | DQ164937.1 | O | ME-SA | PanAsia |  | <https://doi.org/10.3201/eid1112.050908> |
| SKR/2000 | AJ539139.1 | O | ME-SA | PanAsia |  | <https://doi.org/10.1023/B:VIRU.0000032789.31134.eb> |
| MAY/3/2000 | HQ632768.1 | O | ME-SA | PanAsia |  | <https://doi.org/10.1007/s11262-011-0599-3> |
| O/TAI/2/2003 | DQ164980.1 | O | ME-SA | PanAsia |  | <https://doi.org/10.3201/eid1112.050908> |
| O/TUR/8/2000 | DQ164984.1 | O | ME-SA | PanAsia |  | <https://doi.org/10.3201/eid1112.050908> |
| O/TUR/2/2001 | DQ164985.1 | O | ME-SA | PanAsia |  | <https://doi.org/10.3201/eid1112.050908> |
| O/VIT/8/2004 | HQ116275.1 | O | ME-SA | PanAsia |  | <https://doi.org/10.1016/j.meegid.2010.11.003> |
| BHU 27/2004 | HQ268524.1 | O | ME-SA | PanAsia-2 |  | <https://doi.org/10.1093/molbev/msab172> |
| MAY/1/2004 | HQ632770.1 | O | ME-SA | PanAsia-2 |  | <https://doi.org/10.1093/molbev/msab172> |
| PAK/4/2017 | MH784404.1 | O | ME-SA | PanAsia-2 |  | <https://doi.org/10.1128/MRA.01397-18> |
| PAK/37/2010 | MT443600.1 | O | ME-SA | PanAsia-2 |  | <https://doi.org/10.1093/molbev/msab172> |
| SAU/1/2009 | MT443788.1 | O | ME-SA | PanAsia-2 |  | <https://doi.org/10.1093/molbev/msab172> |
| KUW/1/2016 | MT443517.1 | O | ME-SA | PanAsia-2 |  | <https://doi.org/10.1093/molbev/msab172> |
| PAK/20/2010 | MT443590.1 | O | ME-SA | PanAsia-2 |  | <https://doi.org/10.1093/molbev/msab172> |
| SAU/1/2009 | MT443788.1 | O | ME-SA | PanAsia-2 |  | <https://doi.org/10.1093/molbev/msab172> |
| IRN/8/2010 | MT443201.1 | O | ME-SA | PanAsia-2 |  | <https://doi.org/10.1093/molbev/msab172> |
| IRN/7/2015 | MT443385.1 | O | ME-SA | PanAsia-2 |  | <https://doi.org/10.1093/molbev/msab172> |
| ISR/1/2014 | MT443482.1 | O | ME-SA | PanAsia-2 |  | <https://doi.org/10.1093/molbev/msab172> |
| TUR/10/2010 | MT443831.1 | O | ME-SA | PanAsia-2 |  | <https://doi.org/10.1093/molbev/msab172> |
| TUR/12/2013 | MT443859.1 | O | ME-SA | PanAsia-2 |  | <https://doi.org/10.1093/molbev/msab172> |
| SAU/18/2016 | MT443794.1 | O | ME-SA | PanAsia-2 | ANT-10 | <https://doi.org/10.1093/molbev/msab172> |
| AFG/1/2014 | MT443022.1 | O | ME-SA | PanAsia-2 | ANT-10 | <https://doi.org/10.1093/molbev/msab172> |
| AFG/17/2013 | MT443017.1 | O | ME-SA | PanAsia-2 | ANT-10 | <https://doi.org/10.1093/molbev/msab172> |
| AFG/35/2017 | MT443045.1 | O | ME-SA | PanAsia-2 | ANT-10 | <https://doi.org/10.1093/molbev/msab172> |
| AFG/15/2017 | MT443041.1 | O | ME-SA | PanAsia-2 | ANT-10 | <https://doi.org/10.1093/molbev/msab172> |
| AFG/11/2016 | MT443032.1 | O | ME-SA | PanAsia-2 | ANT-10 | <https://doi.org/10.1093/molbev/msab172> |
| PAK/26/2013 | MT443695.1 | O | ME-SA | PanAsia-2 | ANT-10 | <https://doi.org/10.1093/molbev/msab172> |
| PAK/70/2011 | MT443669.1 | O | ME-SA | PanAsia-2 | ANT-10 | <https://doi.org/10.1093/molbev/msab172> |
| PAK/2/2011 | MT443661.1 | O | ME-SA | PanAsia-2 | ANT-10 | <https://doi.org/10.1093/molbev/msab172> |
| PAK/10/2016 | MH784403.1 | O | ME-SA | PanAsia-2 | ANT-10 | [https://doi.org/10.1128/mra.01397-18](https://doi.org/10.1093/molbev/msab172) |
| O/NIAB/PUN/PAK/150/2016 | KY659576.1 | O | ME-SA | PanAsia-2 | ANT-10 | [https://doi.org/10.1007/s00705-018-3775-0](https://doi.org/10.1093/molbev/msab172) |
| PD57/2018 | MT918999.1 | O | ME-SA | SA-2018 |  | <https://doi.org/10.1111/tbed.13954> |
| PD42/2018 | MT918995.1 | O | ME-SA | SA-2018 |  | <https://doi.org/10.1111/tbed.13954> |
| PD325/2018 | MT919005.1 | O | ME-SA | SA-2018 |  | <https://doi.org/10.1111/tbed.13954> |
| PD45/2018 | MT918997.1 | O | ME-SA | SA-2018 |  | <https://doi.org/10.1111/tbed.13954> |
| IC231/2018 | MT936115.1 | O | ME-SA | SA-2018 |  | <https://doi.org/10.1111/tbed.13954> |
| IC207/2018 | MT936111.1 | O | ME-SA | SA-2018 |  | <https://doi.org/10.1111/tbed.13954> |
| IC218/2018 | MT936113.1 | O | ME-SA | SA-2018 |  | <https://doi.org/10.1111/tbed.13954> |
| IC202/2018 | MT936110.1 | O | ME-SA | SA-2018 |  | <https://doi.org/10.1111/tbed.13954> |
| UGA/5/96 | AJ296327.1 | O | EA-1 | EA-1 |  | <https://doi.org/10.1016/j.meegid.2013.04.027> |
| O/KEN/5/2002 | DQ165073.1 | O | EA-2 |  |  | <https://doi.org/10.1111/tbed.12834> |
| TAN/2/2004 | KF561679.1 | O | EA-2 |  |  | <https://doi.org/10.3201/eid1112.050908> |
| O/Fayoum/EGY/2014 | KR261670.1 | O | EA-3 |  |  | <https://doi.org/10.3201/eid1112.050908> |
| O/Qaliubia/EGY/2013 | KR261668.1 | O | EA-3 |  |  | <https://doi.org/10.1111/tbed.12200> |
| ETH/60/2005 | FJ798143.1 | O | EA-4 |  |  | <https://doi.org/10.14202%2Fvetworld.2019.190-197> |
| CAR/16/2000 | HM211080.1 | O | WA |  |  | <https://doi.org/10.14202%2Fvetworld.2019.190-197> |
| O/PHI/7/96 | AJ294926.1 | O | CATHAY |  |  | <https://doi.org/10.3201/eid1509.090091> |
| O/HKN/6/83 | AJ294919.1 | O | CATHAY |  |  | <https://doi.org/10.1111/j.1865-1682.2010.01151.x> |
| O/Corrientes/Arg/06 | DQ834727.1 | O | EURO-SA |  |  | <https://doi.org/10.3201/eid1112.050908> |
| O/Chuquisaca/Bol/03 | DQ834712.1 | O | EURO-SA |  |  | <https://doi.org/10.3201/eid1112.050908> |
| O/HongKong/P424/2011 | JF968193.1 | O | SEA |  |  | <https://doi.org/10.1016/j.virusres.2006.09.006> |
| O/HongKong/P404/2010 | JF968186.1 | O | SEA |  |  | <https://doi.org/10.1016/j.virusres.2006.09.006> |
| ISA/8/83 | AJ303503.1 | O | ISA-1 |  |  | <http://dx.doi.org/10.1016/j.vetmic.2012.03.016> |
| JAV/5/72 | AJ303509.1 | O | ISA-2 |  |  | <http://dx.doi.org/10.1016/j.vetmic.2012.03.016> |
| IND 101-99 | DQ989310.1 | Asia 1 |  |  |  | <https://doi.org/10.1016/j.virusres.2008.04.010> |
| BAN GA Sa-197 2013 | KJ754939.1 | A | ASIA | G-VII |  | <https://doi.org/10.1128/genomeA.00506-14> |
| O/India/R2/75 (field vaccine strain) | AF204276.1 | O |  |  |  | <https://doi.org/10.1016/j.vaccine.2014.11.058> |
| BAN/MY/My-466/2021 | OP320455.1 | O | ME-SA | SA-2018 | MYMBD21 | <https://doi.org/10.1038/s41598-023-36830-w> |
| BAN/MY/My-469/2021 | OP320456.1 | O | ME-SA | SA-2018 | MYMBD21 | <https://doi.org/10.1038/s41598-023-36830-w> |
| BAN/MY/My-478/2021 | OP320457.1 | O | ME-SA | SA-2018 | MYMBD21 | <https://doi.org/10.1038/s41598-023-36830-w> |

**Supplementary Table S4 Complete Genome Annotation of BAN/MY/My-466/2021**

| Gene fragments | Nucleotide range | Nucleotide length (bp) | Amino acid residue |
| --- | --- | --- | --- |
| 5ꞌ UTR | 1-1100 | 1100 | -- |
| Lpro | 1101-1703 | 603 | 201 |
| VP4 | 1704-1958 | 255 | 85 |
| VP2 | 1959-2612 | 654 | 218 |
| VP3 | 2613-3272 | 660 | 220 |
| VP1 | 3273-3911 | 639 | 213 |
| 2A | 3912-3959 | 48 | 16 |
| 2B | 3960-4421 | 462 | 154 |
| 2C | 4422-5375 | 954 | 318 |
| 3A | 5376-5834 | 459 | 153 |
| 3B | 5835-6047 | 213 | 71 |
| 3C | 6048-6686 | 639 | 213 |
| 3D | 6687-8096 | 1410 | 470 |
| 3ꞌ UTR | 8097-8216 | ~120 | -- |


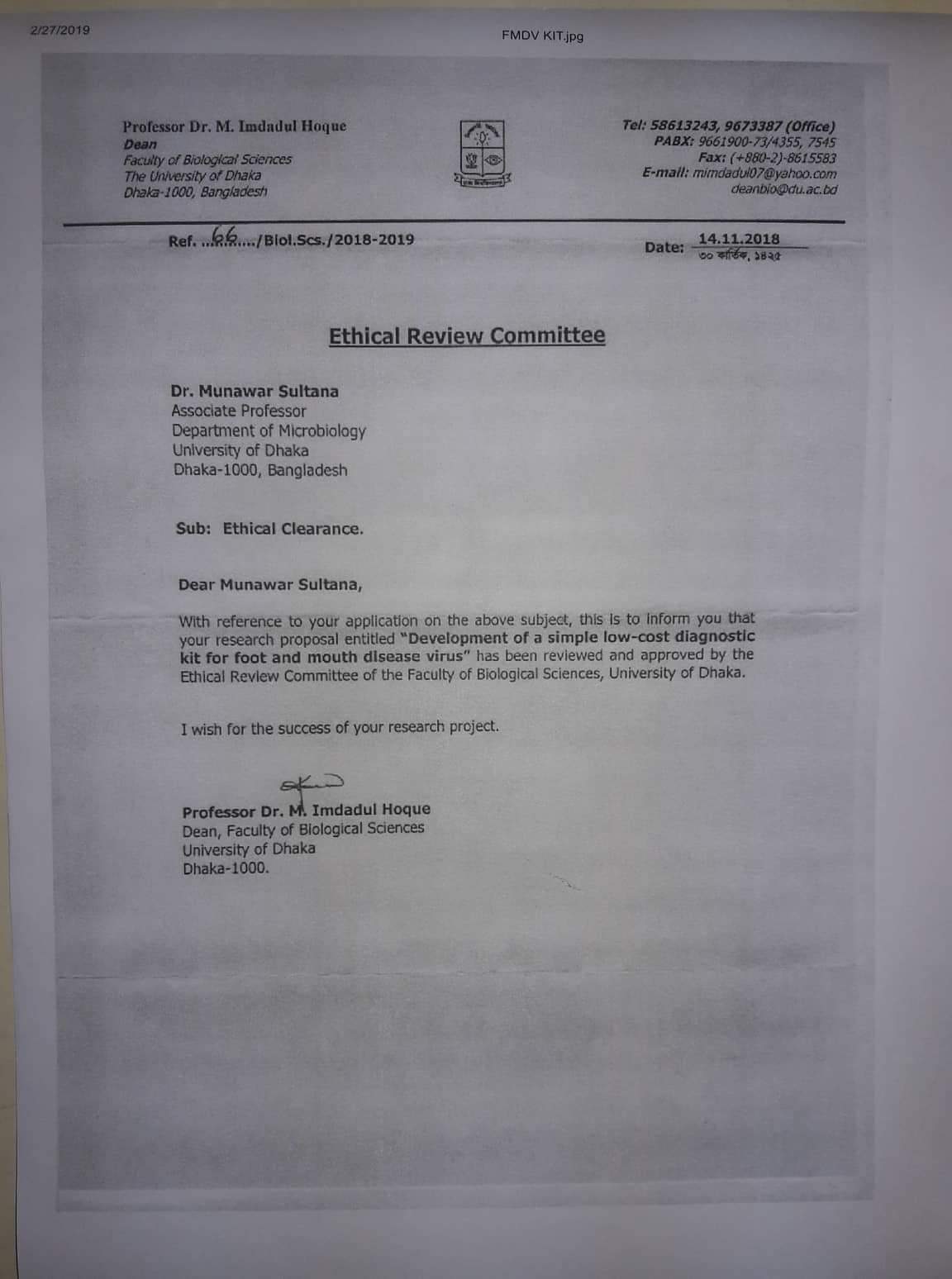


**Supplementary Figure S1. Ethical Clearance for the experiment (Ref:66/Biol. Sci./2018-19; Date: 14-11-2018).**


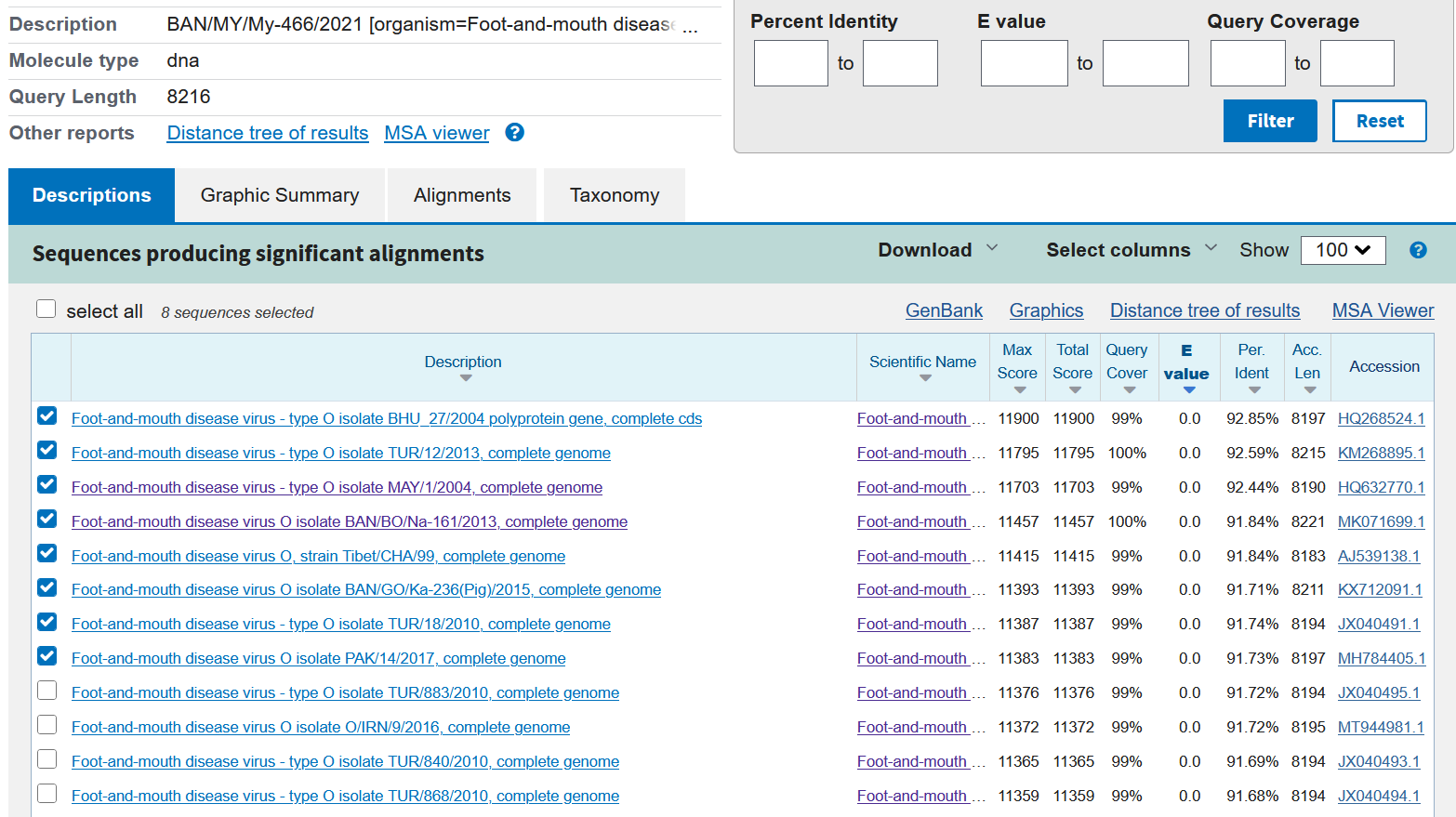


**Supplementary Figure S2. BLAST search result of BAN/MY/My-446/2021 complete genome.**


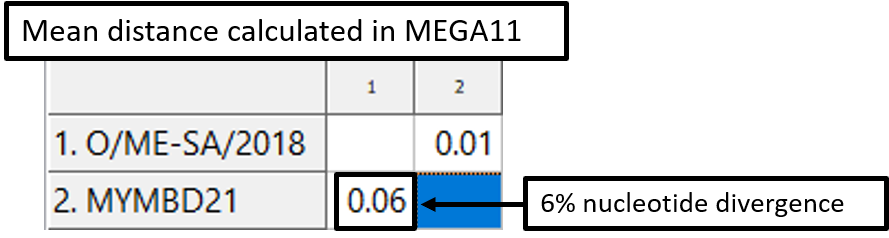


**Supplementary Figure S3. Mean distance between O/ME-SA/SA-2018 lineage and BAN/MY/MY-466/2021 (MYMBD21) isolate.**

**
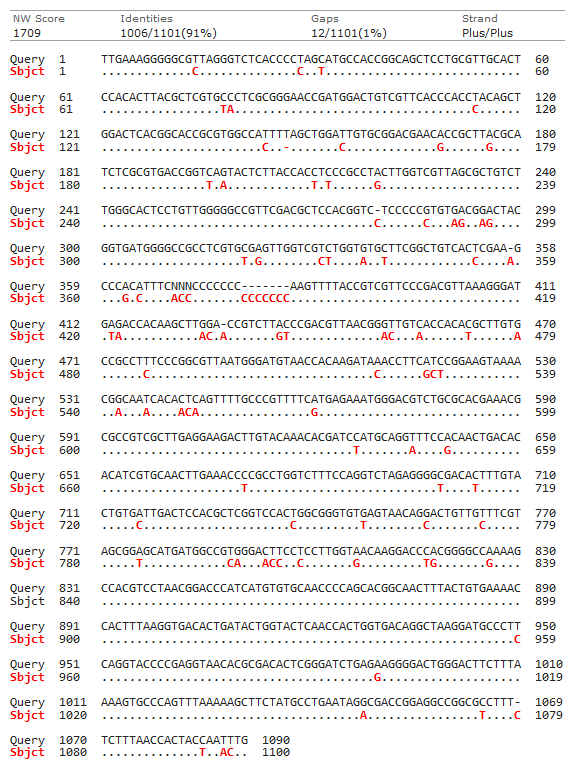
**

**Supplementary Figure S4. Alignment of 5ꞌ UTR of BAN/MY/My-466/2021 and Reference sequence (BHU_27/2004).** (Query=Reference sequence, Subject=BAN/MY/My-466/2021). 91% identity was detected.


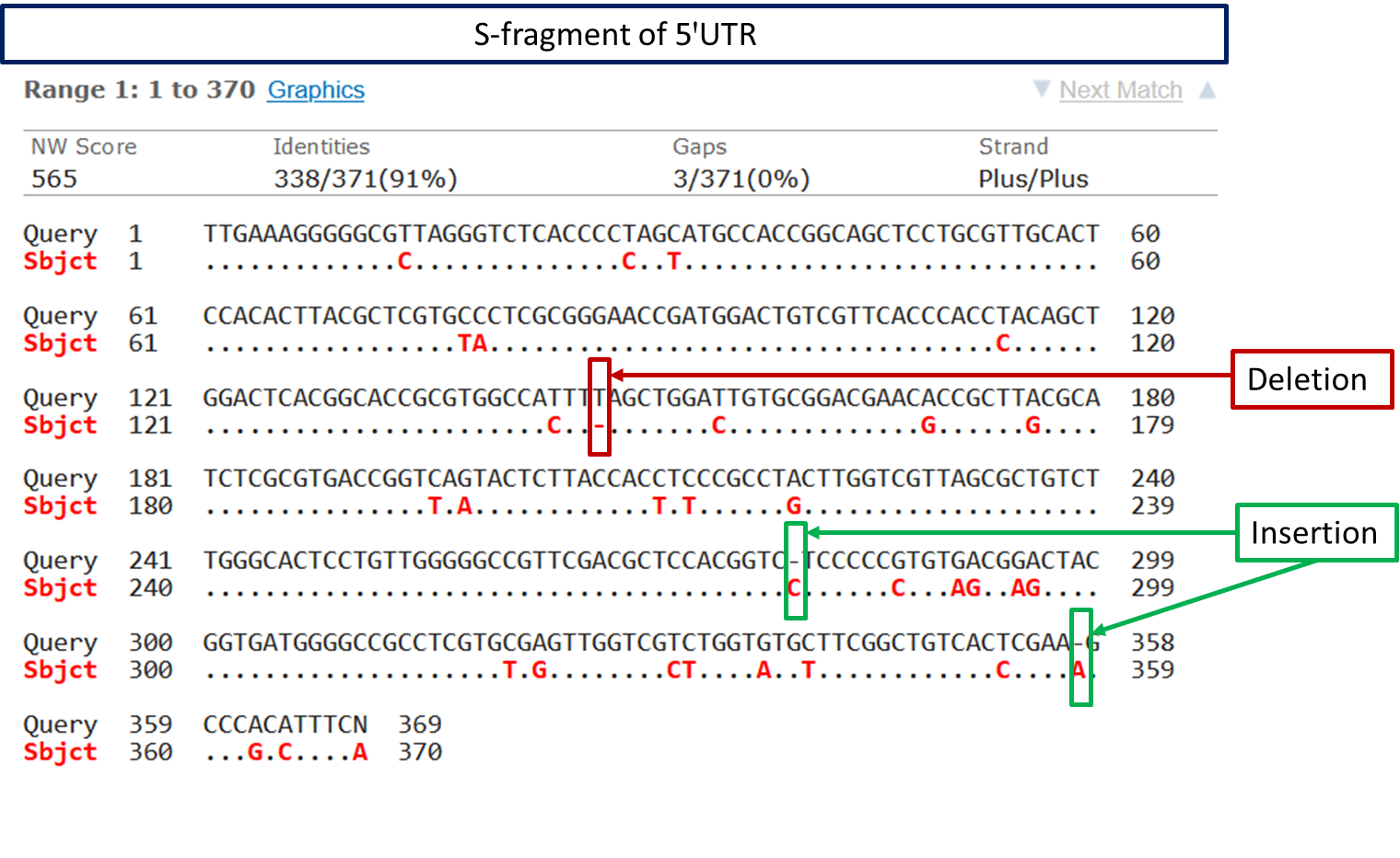


**Supplementary Figure S5. Alignment of S-fragment of 5ꞌ UTR between BAN/MY/My-466/2021 and Reference sequence (BHU_27/2004).** (Query=Reference sequence, Subject=BAN/MY/My-466/2021). 30 amino acid substitutions, 1 nucleotide deletion, and 2 nucleotide insertions were detected.


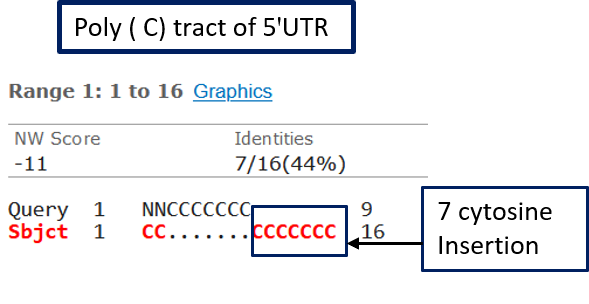


**Supplementary Figure S6. Alignment of poly (C) tract of 5ꞌ UTR of BAN/MY/My-466/2021 and Reference sequence (BHU_27/2004).** (Query=Reference sequence, Subject=BAN/MY/My-466/2021). Insertion of seven cytosines in BAN/MY/My-466/2021 poly (C) was detected.

Insertion

Deletion


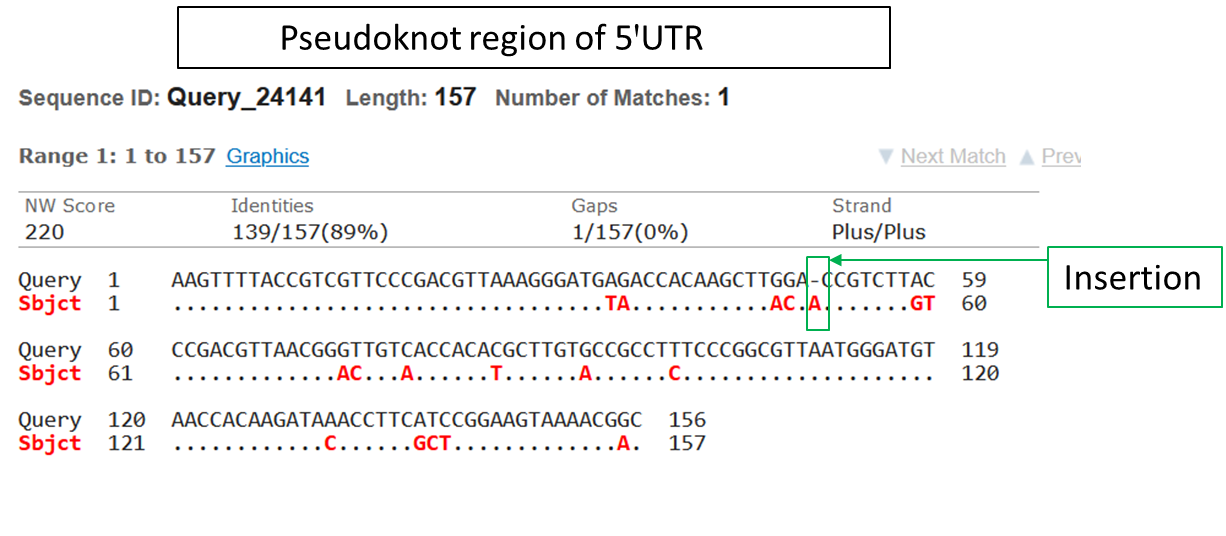


**Supplementary Figure S7. Alignment of pseudoknot region of 5ꞌ UTR of BAN/MY/My-466/2021 and Reference sequence (BHU_27/2004).** (Query=Reference sequence, Subject=BAN/MY/My-466/2021). 17 substitutions and 1 nucleotide insertion were detected.


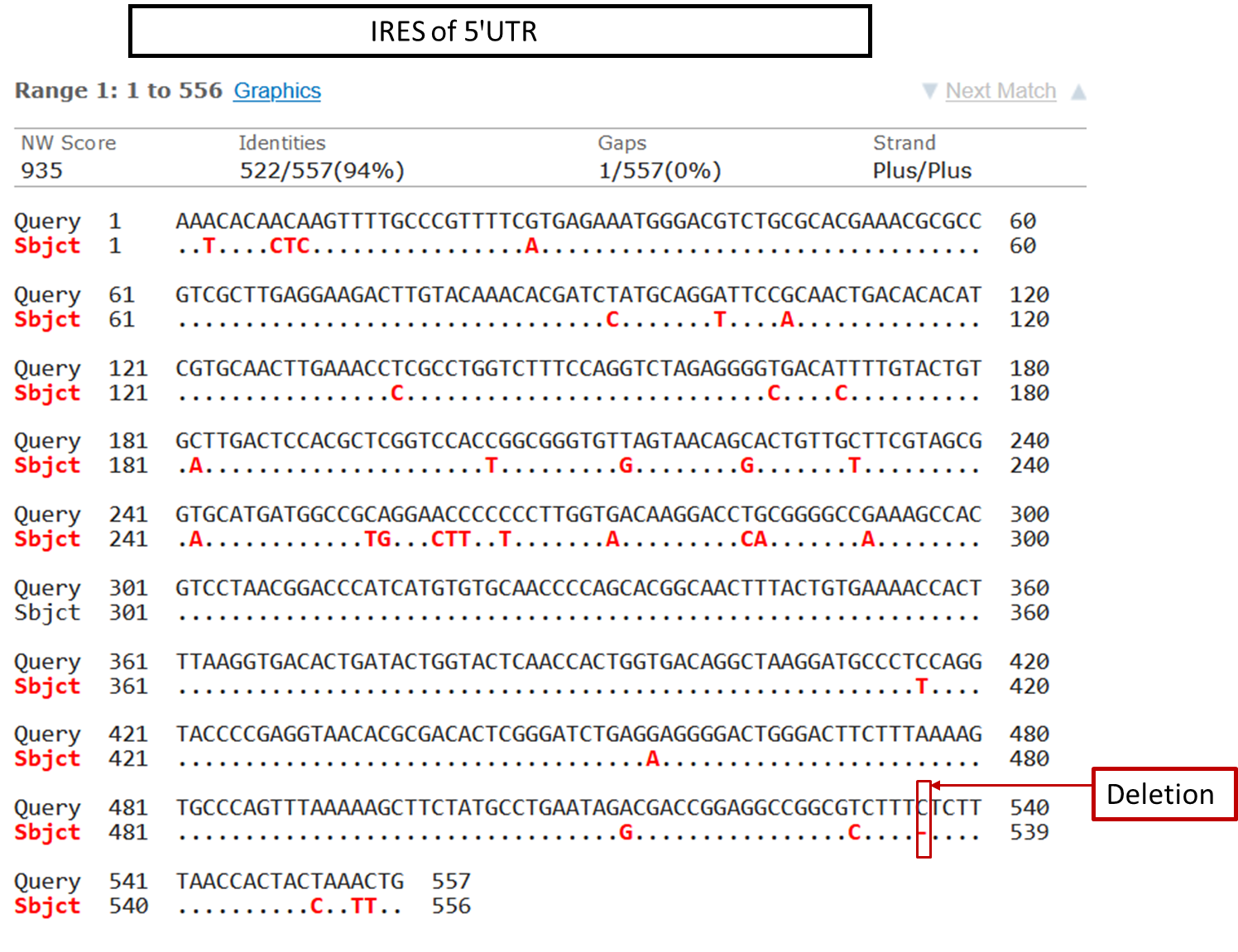


**Supplementary Figure S8. Alignment of IRES (Inter Ribosome Entry Site) region of 5ꞌ UTR of BAN/MY/My-466/2021 and Reference sequence (BHU_27/2004).** (Query=Reference sequence, Subject=BAN/MY/My-466/2021). 34 substitutions and 1 nucleotide deletion were detected.


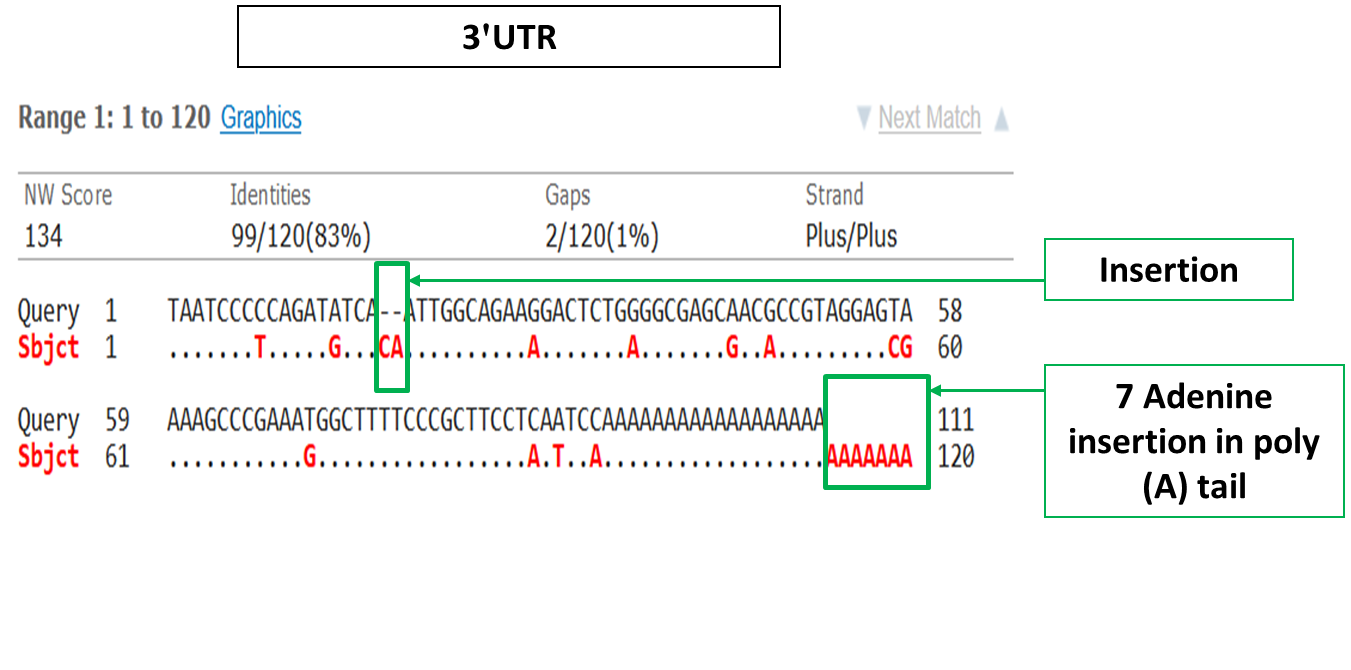


**Supplementary Figure S9. Alignment of 3ꞌ UTR of BAN/MY/My-466/2021 and Reference sequence (BHU_27/2004).** (Query=Reference sequence, Subject=BAN/MY/My-466/2021). 2 nucleotides insertions and 7 adenines insertion in poly (A) tail were detected.


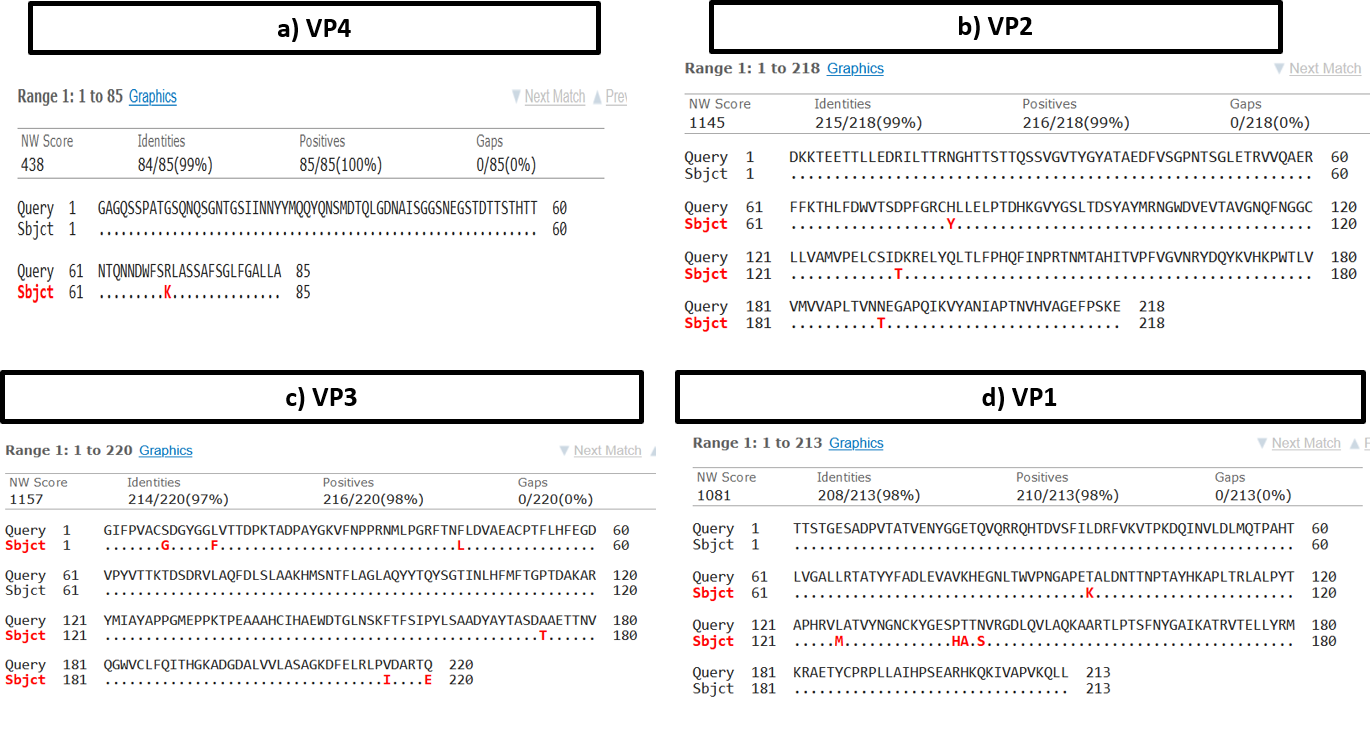


**Supplementary Figure S10. Alignment of a) VP4, b) VP2, c) VP3, d) VP1 proteins of BAN/MY/My-466/2021 and Reference sequence (BHU_27/2004).** (Query=Reference sequence, Subject=BAN/MY/My-466/2021).


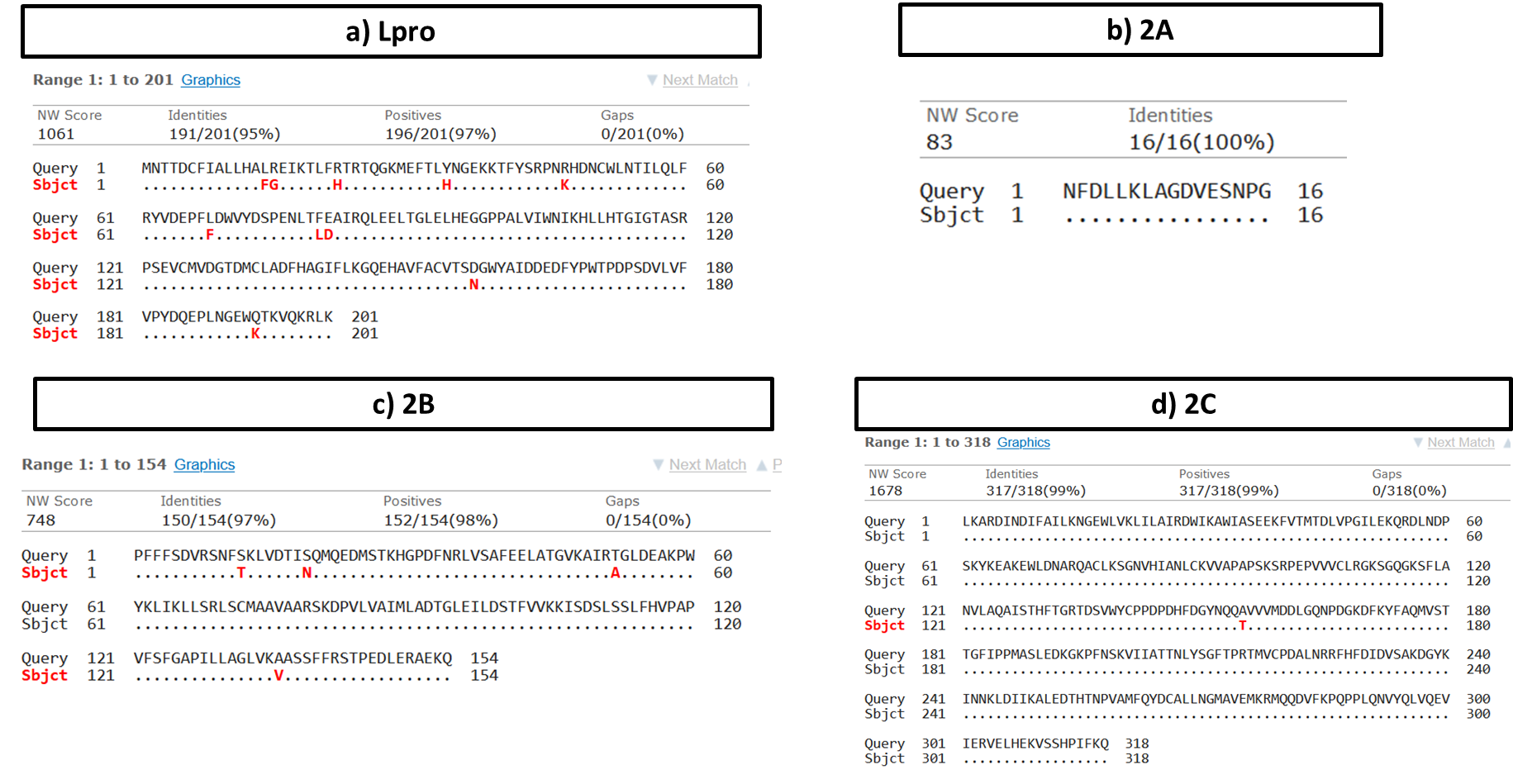


**Supplementary Figure S11. Alignment of a) Lpro, b) 2A, c) 2B, d) 2C encoded proteins of BAN/MY/My-466/2021 and Reference sequence (BHU_27/2004).** (Query=Reference sequence, Subject=BAN/MY/My-466/2021).


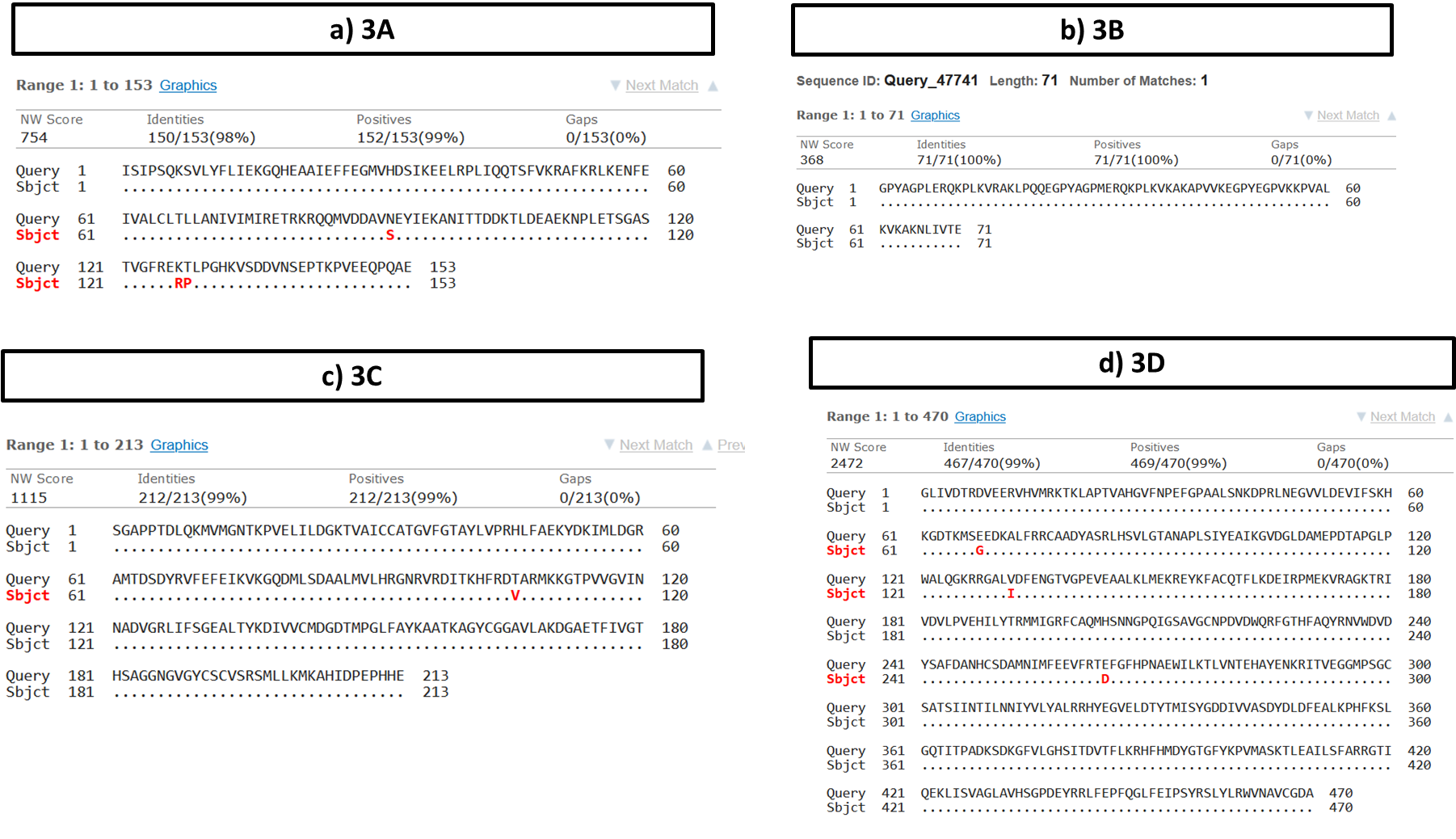


**Supplementary Figure S12. Alignment of a) 3A, b) 3B, c) 3C, d) 3D encoded proteins of BAN/MY/My-466/2021 and Reference sequence (BHU_27/2004).** (Query=Reference sequence, Subject=BAN/MY/My-466/2021).

**
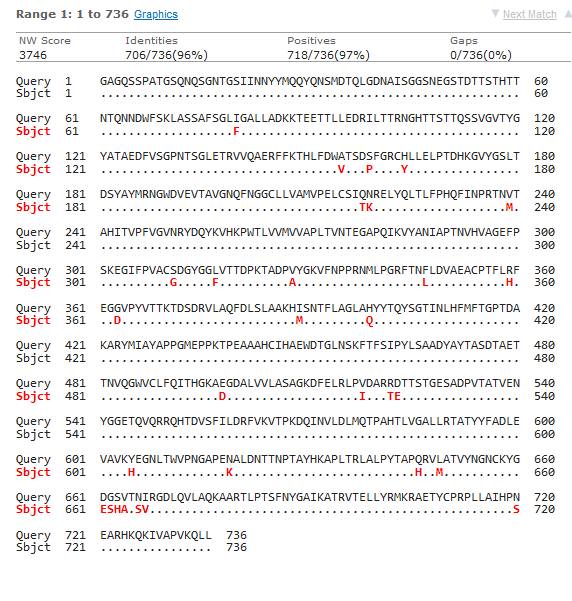
 Supplementary Figure S13. Alignment of full capsid protein sequence of BAN/MY/My-466/2021 against field vaccine strain (O/India/R2/75). (Query= O/India/R2/75, Subject=BAN/MY/My-466/2021)**. 96% protein identity was detected.


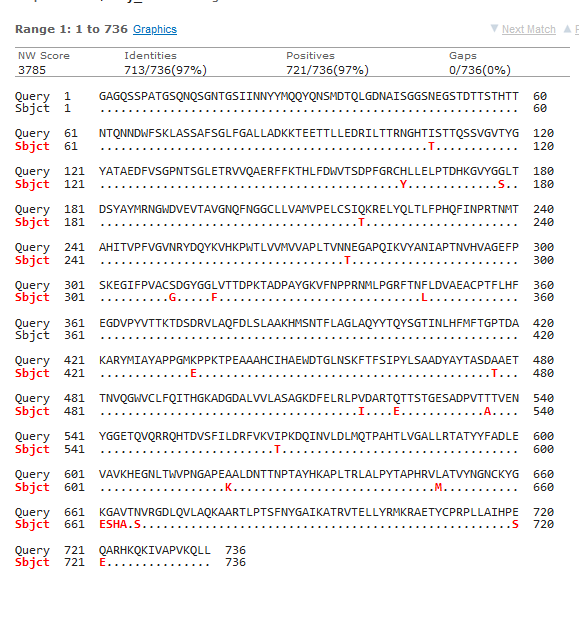


**Supplementary Figure S14. Alignment of full capsid protein sequence of BAN/MY/My-466/2021 against proposed local vaccine strain (BAN/TA/DH-301/2016). (Query= BAN/TA/DH-301/2016, Subject=BAN/MY/My-466/2021)**. 97% protein identity was detected.


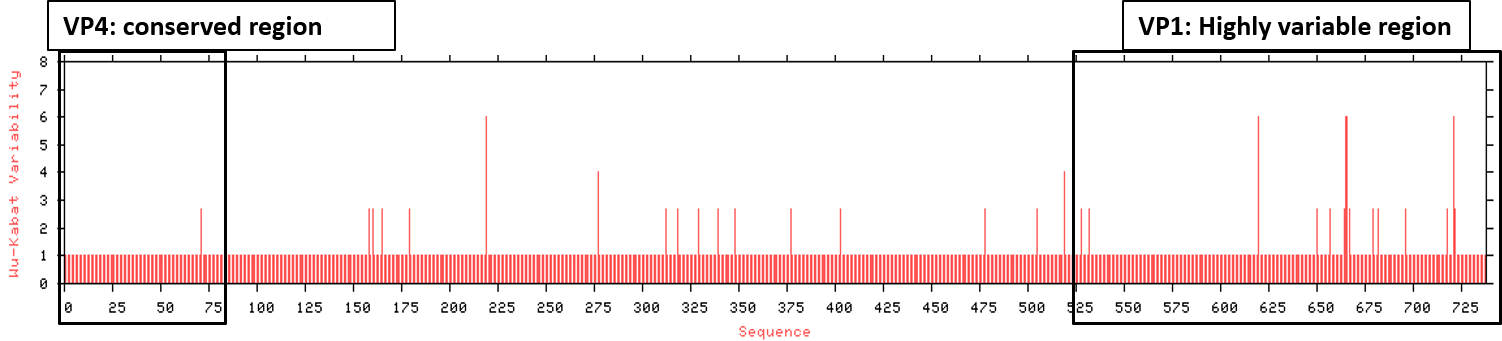


**Supplementary Figure S15. Wu-Kabat Protein variability plot of BAN/MY/My-466/2021 capsid region against field and proposed local vaccine strains.** VP4 (1-85), VP2 (86-303), VP3 (304-523), VP1(524-736).


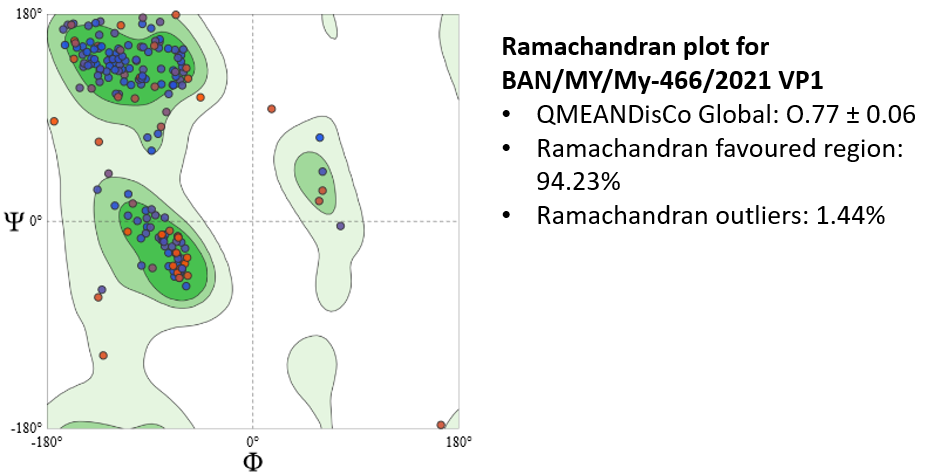


**Supplementary Figure S16. Ramachandran plot for VP1 3D model of BAN/MY/My-466/2021.**


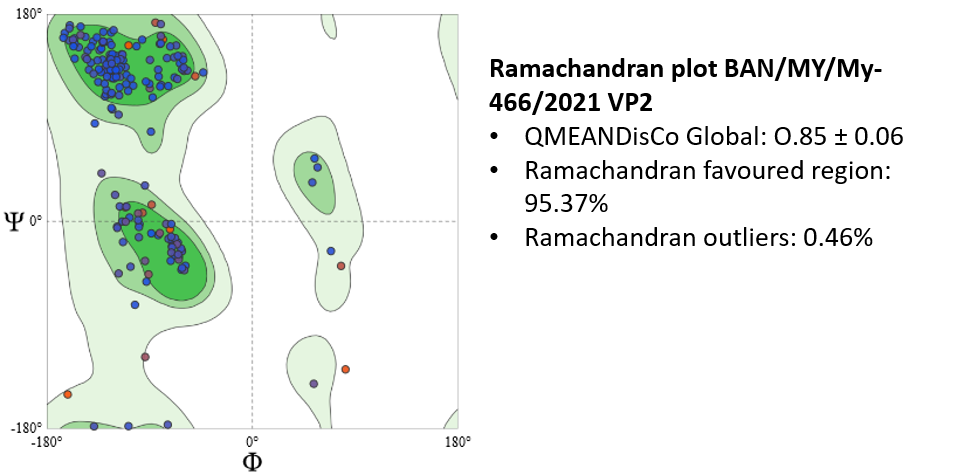


**Supplementary Figure S17. Ramachandran plot for VP2 3D model of BAN/MY/My-466/2021.**


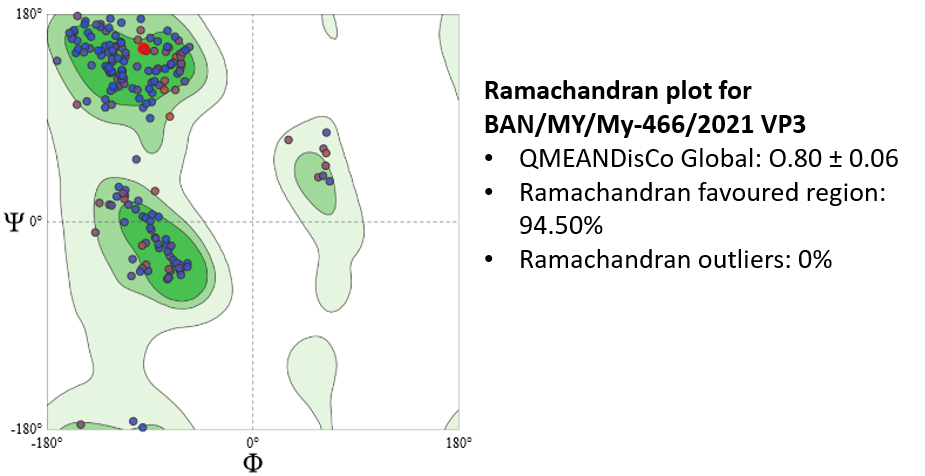


**Supplementary Figure S18. Ramachandran plot for VP3 3D model of BAN/MY/My-466/2021.**


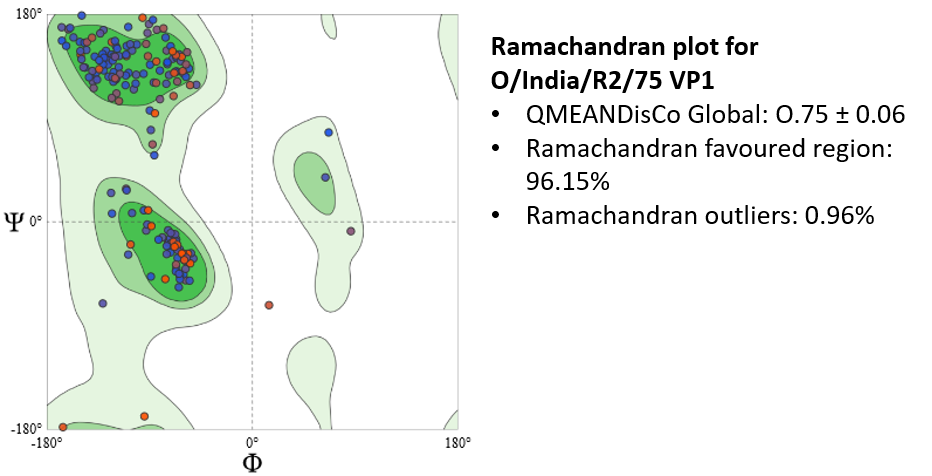


**Supplementary Figure S19. Ramachandran plot for VP1 3D model of O/India/R2/75.**


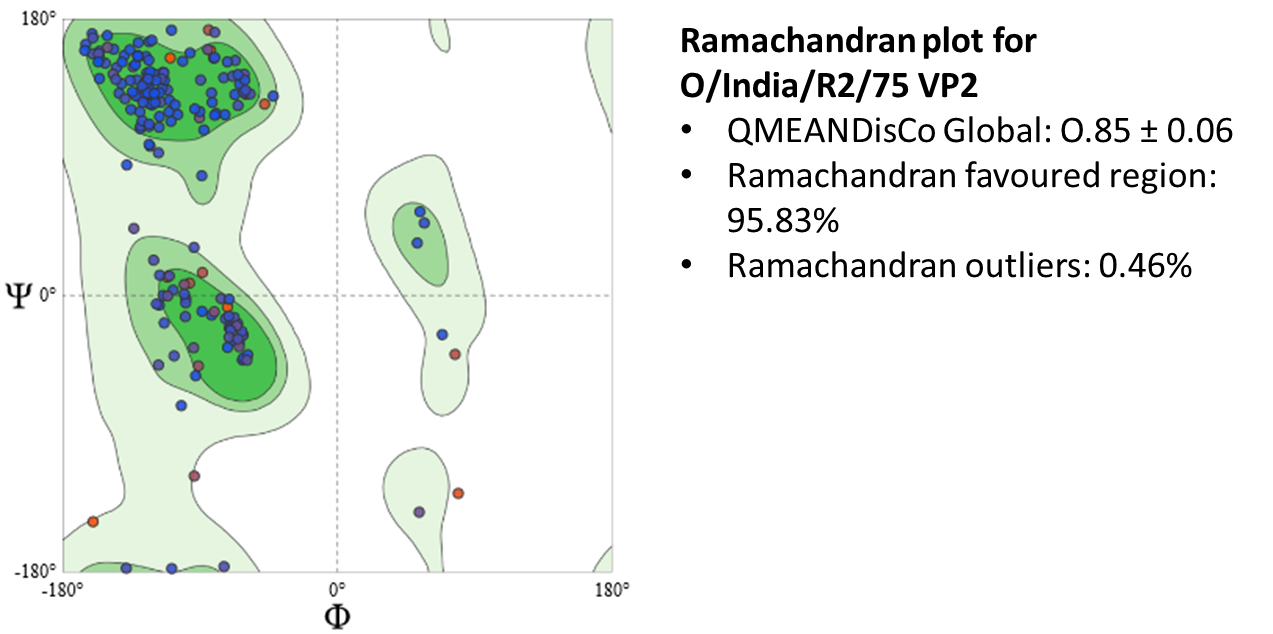


**Supplementary Figure S20. Ramachandran plot for VP2 3D model of O/India/R2/75.**


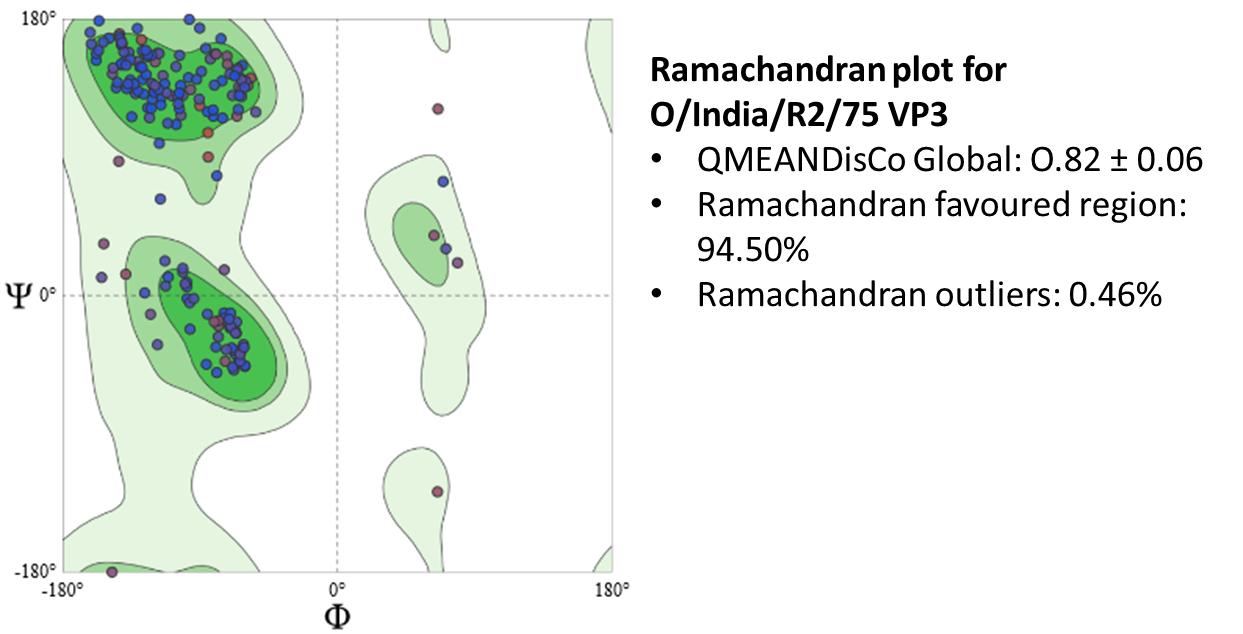


**Supplementary Figure S21. Ramachandran plot for VP3 3D model of O/India/R2/75.**


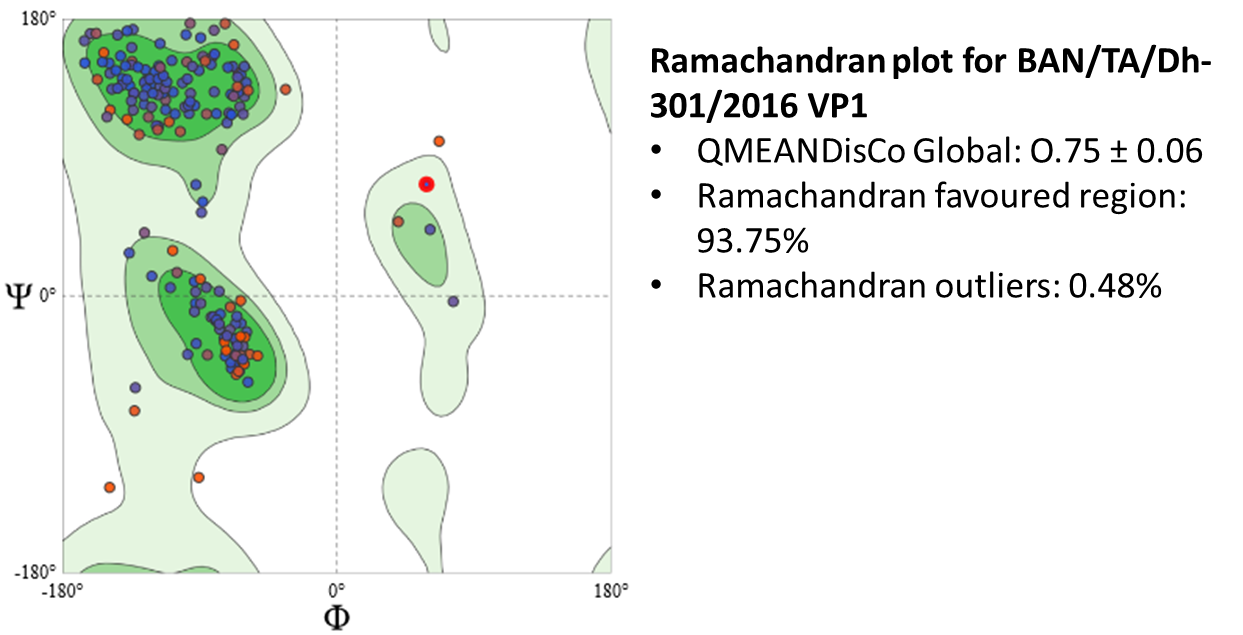


**Supplementary Figure S22. Ramachandran plot for VP1 3D model of BAN/TA/Dh-301/2016.**

**
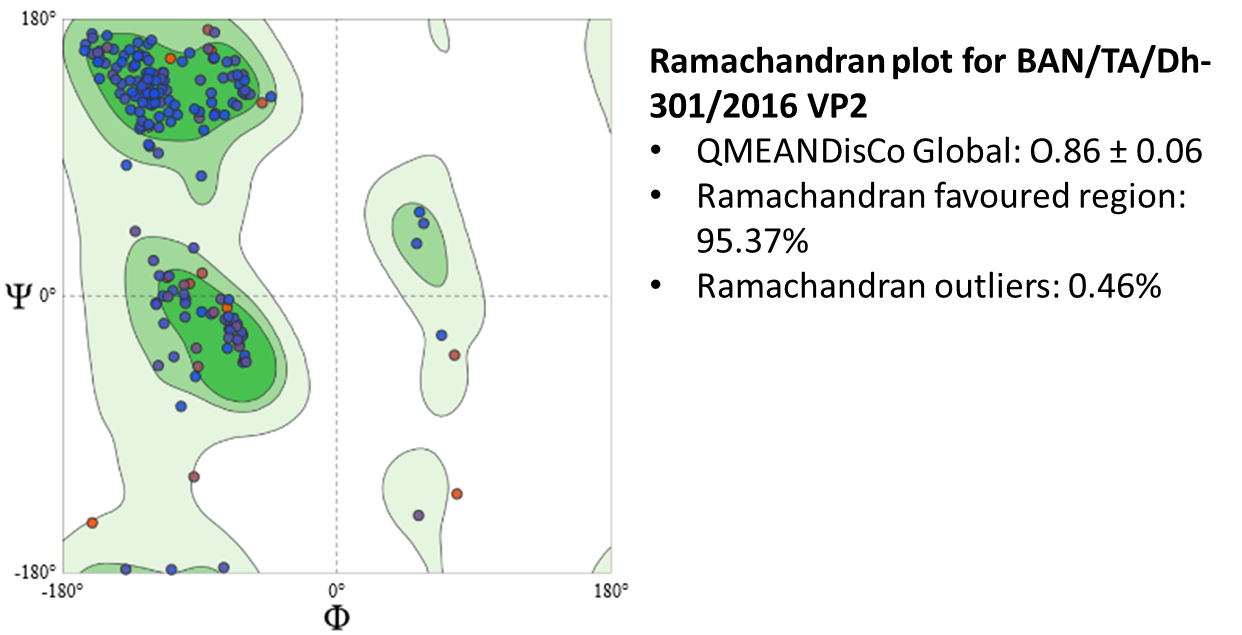
**

**Supplementary Figure S23. Ramachandran plot for VP2 3D model of BAN/TA/Dh-301/2016.**

**
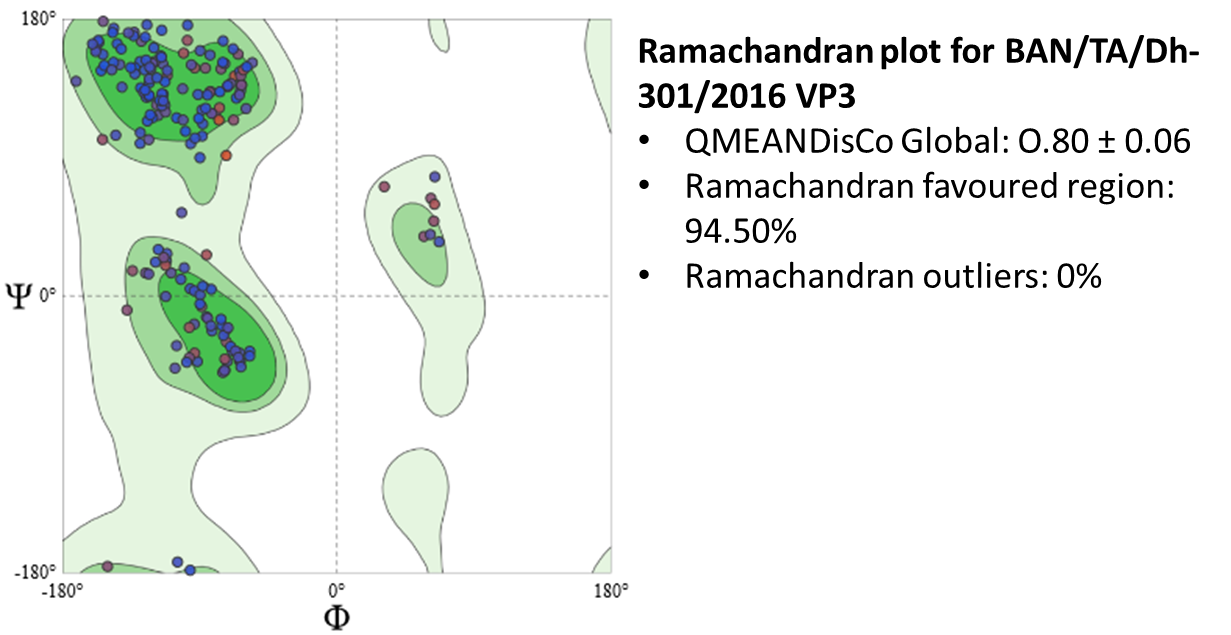
**

**Supplementary Figure S24. Ramachandran plot for VP3 3D model of BAN/TA/Dh-301/2016.**

**
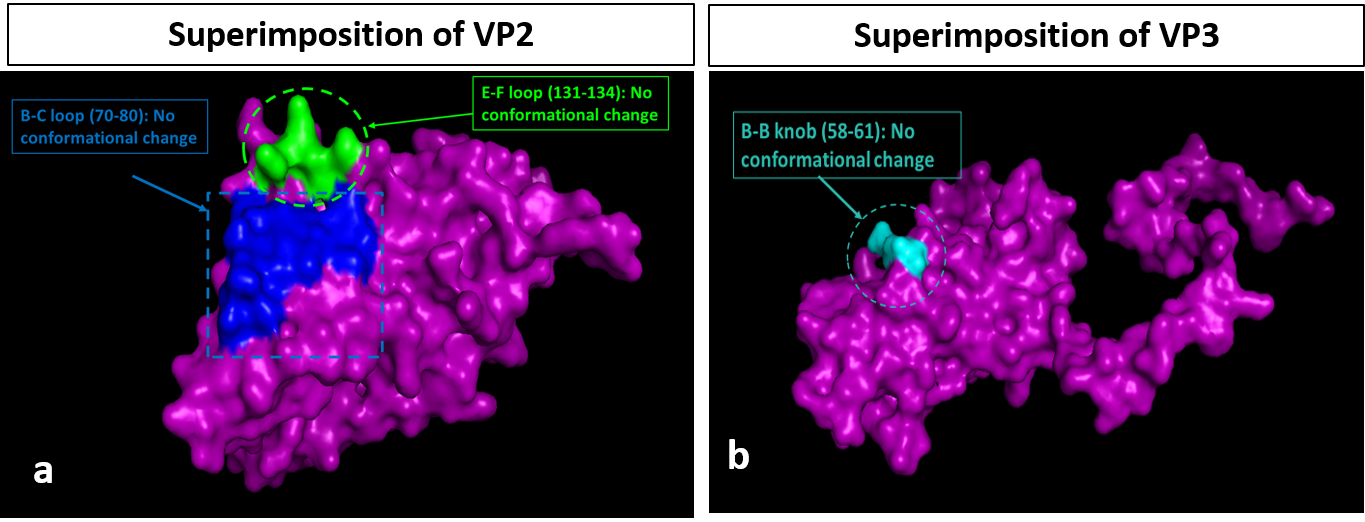
**

**Supplementary Figure S25. Superimposed 3D model against field vaccine strain (O/India/R2/75).**

**VP2 and VP3 of vaccine strain was presented in surface style (purple) and BAN/MY/My-466/2021 was presented in cartoon style that cannot be seen as no conformational change was observed.**

1. **VP2 of BAN/MY/My-466/2021 superimposed on VP2 of field vaccine (O/India/R2/75): B-C loop (blue), E-F loop (light green). No conformational change was observed.**
2. **VP3 of BAN/MY/My-466/2021 superimposed on VP3 of field vaccine (O/India/R2/75). B-B knob (cyan). No conformational change was observed.**


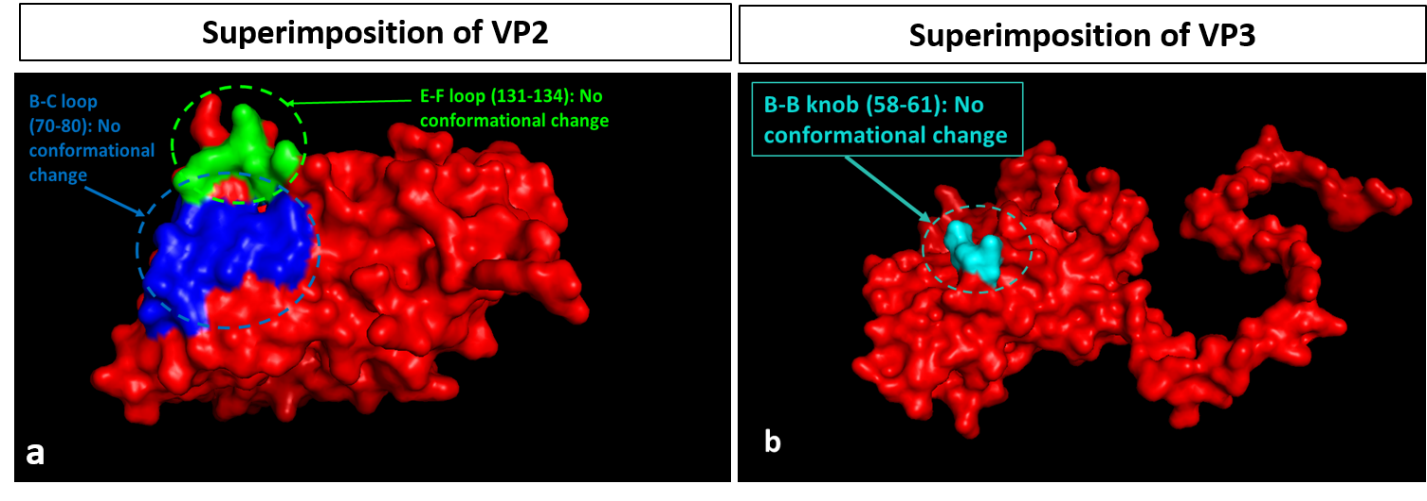


**Supplementary Figure S26. Superimposed 3D model against proposed local vaccine strain (BAN/TA/Dh-301/2016). VP2 and VP3 of vaccine strain was presented in surface style (red) and BAN/MY/My-466/2021 was presented in cartoon style.**

1. **VP2 of BAN/MY/My-466/2021 superimposed on VP2 of proposed local vaccine (BAN/TA/Dh-301/2016): B-C loop (blue), E-F loop (light green). No conformational change was observed.**
2. **VP3 of BAN/MY/My-466/2021 superimposed on VP3 of proposed local vaccine (BAN/TA/Dh-301/2016): B-B knob (cyan). No conformational change was observed.**

**References:**

Abdul-Hamid NF, Fırat-Saraç M, Radford AD *et al.* Comparative sequence analysis of representative foot-and-mouth disease virus genomes from Southeast Asia. *Virus Genes* 2011;**43**:41–5.

Ali MR, Ullah H, Siddique MA *et al.* Complete Genome Sequence of Pig-Originated Foot-and-Mouth Disease Virus Serotype O from Bangladesh. *Genome Announc* 2016;**4**:e01150-16.

Hossain K.A., Anjume H., Alam K.M.M. *et al.* Evidence of O/ME-SA/2018 lineage of Foot-and-Mouth Disease Virus serotype O in Bangladesh with a mutational trend of emerging novel sublineage, MYMBD21. 2022.

Hossain KA, Anjume H, Alam KMM *et al.* Emergence of a novel sublineage, MYMBD21 under SA-2018 lineage of Foot-and-Mouth Disease Virus serotype O in Bangladesh. *Sci Rep* 2023;**13**:9817.

Ma X, Li P, Bai X *et al.* Sequences outside that of residues 93–102 of 3A protein can contribute to the ability of foot-and-mouth disease virus (FMDV) to replicate in bovine-derived cells. *Virus Research* 2014;**191**:161–71.

Reid SM, Ferris NP, Hutchings GH *et al.* Primary diagnosis of foot-and-mouth disease by reverse transcription polymerase chain reaction. *Journal of Virological Methods* 2000;**89**:167–76.

Samuel AR, Knowles NJ. Foot-and-mouth disease type O viruses exhibit genetically and geographically distinct evolutionary lineages (topotypes). *Journal of General Virology* 2001;**82**:609–21.

Sanyal A, Mohapatra JK, Kumar RM *et al.* Complete nucleotide sequence analysis of a vaccine strain and a field isolate of foot-and-mouth disease virus serotype Asia1 with an insertion in VP1 genomic region. *Acta Virol* 2004;**48**:159–66.

Sultana M, Siddique MA, Momtaz S *et al.* Complete Genome Sequence of Foot-and-Mouth Disease Virus Serotype O Isolated from Bangladesh. *Genome Announc* 2014;**2**:e01253-13.
